# Hybrid laser precision engineering of transparent hard materials: challenges, solutions and applications

**DOI:** 10.1038/s41377-021-00596-5

**Published:** 2021-08-05

**Authors:** Huagang Liu, Wenxiong Lin, Minghui Hong

**Affiliations:** 1 Department of Electrical and Computer Engineering, National University of Singapore, 4 Engineering Drive 3, 117576 Singapore, Singapore; 2 Key Laboratory of Optoelectronic Materials Chemistry and Physics, Fujian Institute of Research on the Structure of Matter, Chinese Academy of Sciences, Fuzhou, 350002 China

**Keywords:** Laser material processing, Laser-produced plasmas

## Abstract

Laser has been demonstrated to be a mature and versatile tool that presents great flexibility and applicability for the precision engineering of a wide range of materials over other established micromachining techniques. Past decades have witnessed its rapid development and extensive applications ranging from scientific researches to industrial manufacturing. Transparent hard materials remain several major technical challenges for conventional laser processing techniques due to their high hardness, great brittleness, and low optical absorption. A variety of hybrid laser processing technologies, such as laser-induced plasma-assisted ablation, laser-induced backside wet etching, and etching assisted laser micromachining, have been developed to overcome these barriers by introducing additional medium assistance or combining different process steps. This article reviews the basic principles and characteristics of these hybrid technologies. How these technologies are used to precisely process transparent hard materials and their recent advancements are introduced. These hybrid technologies show remarkable benefits in terms of efficiency, accuracy, and quality for the fabrication of microstructures and functional devices on the surface of or inside the transparent hard substrates, thus enabling widespread applications in the fields of microelectronics, bio-medicine, photonics, and microfluidics. A summary and outlook of the hybrid laser technologies are also highlighted.

## Abstract (web summary)

Schematic of the dynamic process of the laser-induced plasma assisted ablation. The process consists of laser heating, plasma generation, absorption enhancement and material removal.

## Graphical abstract

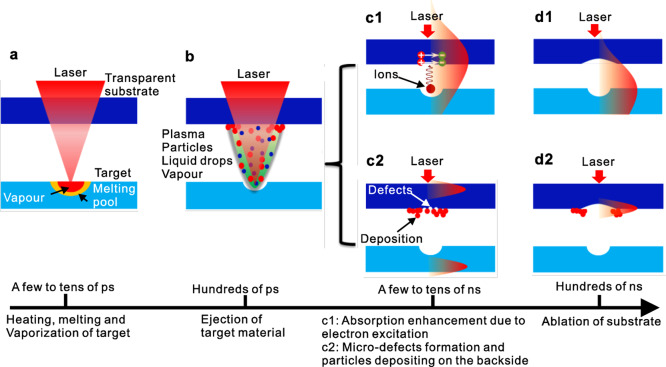

## Introduction

Transparent hard materials, including diamond, sapphire, and glass, are a special group of materials that allow light to pass through without energy loss from scattering or absorption. Excellent properties, such as high hardness, high chemical stability, and wide-band transparency, make these transparent materials attractive in varieties of particular applications ranging from industry, scientific research to daily life^1–7^. On the other hand, these properties also make them difficult to be processed mechanically or chemically. It is a long-time technical challenge on how to find suitable engineering tools to fabricate devices and structures precisely on these transparent hard substrates, especially at the microscale. Laser processing is a light-energy-based engineering technology that has been widely used for cutting, drilling, marking, welding, surface patterning, and structuring of almost all kinds of materials^8–14^. The wide applications of laser processing can be attributed to its various distinct advantages in terms of high efficiency, simple process, and non-contact nature^15–20^. With the unique properties of high coherence and directionality, the laser beam can be focused locally to a small spot size typically down to sub-micron scale, enabling material processing and high-quality device fabrication at micro and nano-level of accuracy^21,22^. However, there are technical challenges on how to process transparent hard substrates precisely for conventional laser engineering: (1) It is generally known that effective laser processing requires a strong absorption of laser energy. Some of the transparent hard materials have large bandgaps at a broadband wavelength range from UV to mid-infrared (IR). It is difficult to accumulate adequate energy for laser ablation or modification because a small amount of pulse energy is absorbed directly; (2) Most transparent materials, such as sapphire and diamond, are extremely brittle and hard to work with. Laser thermal effect and shock wave interaction can induce large temperature gradients and localized mechanical stress. Therefore, stress-induced cracks are easy to develop on the surface or inside the substrate during laser processing. How to prevent or reduce crack formation is one of the key challenges for high-quality laser processing; (3) laser processing for the fabrication of functional devices like optical and microfluidic components often requires a low roughness and high accuracy, which raises a great challenge to the laser processing on how to achieve roughness down to optical quality; (4) low efficiency of point-to-point laser ablation is another factor that inherently limits its practical applications, in particular for transparent materials with low energy absorption.

A variety of techniques have been developed to address these challenges and provide lots of practical solutions for high quality and high precision laser engineering of transparent hard materials. The simplest method is the direct laser ablation. Because the materials are transparent to convenient laser wavelengths, this direct ablation method requires special lasers which are high absorptive to the substrate materials via either linear or nonlinear absorption. For the linear process, laser wavelengths should be beyond the transparency window, such as UV, VUV, mid-IR, and far-IR lasers^23–26^. For the case of nonlinear process, ultrafast lasers are required to generate extremely high peak intensity which can induce nonlinear effects, such as multiphoton ionization and absorption^27–31^. In order to overcome the issue of low energy absorption of long pulse lasers at visible and NIR regions, a variety of hybrid laser processing techniques, like laser-induced backside, laser-induced plasma-assisted ablation (LIPAA)^32^, and laser-induced backside wet etching (LIBWE)^33^, have been developed by introducing additional absorbing media, such as liquids, plasmas, and solid absorber layers. Absorption of the incident laser energy can be significantly enhanced, allowing the widely used industrial available lasers for efficient processing of transparent hard materials. In addition, the hybrid laser processing which combines different processes, such as laser-assisted milling^34^, laser modification followed by wet or dry etching, and laser processing with multiple lasers^35,36^, offers a promising alternative for surface microstructure, even allowing three-dimensional (3D) structures fabrication inside glass substrates. Currently, these novel hybrid laser techniques have been widely applied to laser micro/nano-processing of transparent hard materials, which finds increasing applications in the fields of microelectronics, bio-medicine, optoelectronics, and mechanical engineering.

There have been several review articles available on the topic of ultrafast laser processing^27,37,38^, ultrafast laser micromachining of transparent materials^28,39–41^, laser cutting of glass^42^, and direct laser writing for surface micro/nano-structuring and functional micro-devices fabrication^19,20,22^. This review will focus on the hybrid laser processes for the precision engineering of transparent hard materials. An overview of the characteristics and the recent advancement of these hybrid laser technologies are presented. How these technologies are utilized to address the challenges faced in the laser processing of transparent hard materials and the physical processes behind them are systematically analyzed. The applications of these hybrid processes in both fundamental research and industrial fields are discussed. Finally, a summary and outlook of these technologies are highlighted.

## Laser-induced plasma-assisted ablation

### Characteristics and methodology of LIPAA

**Fig. 1:**
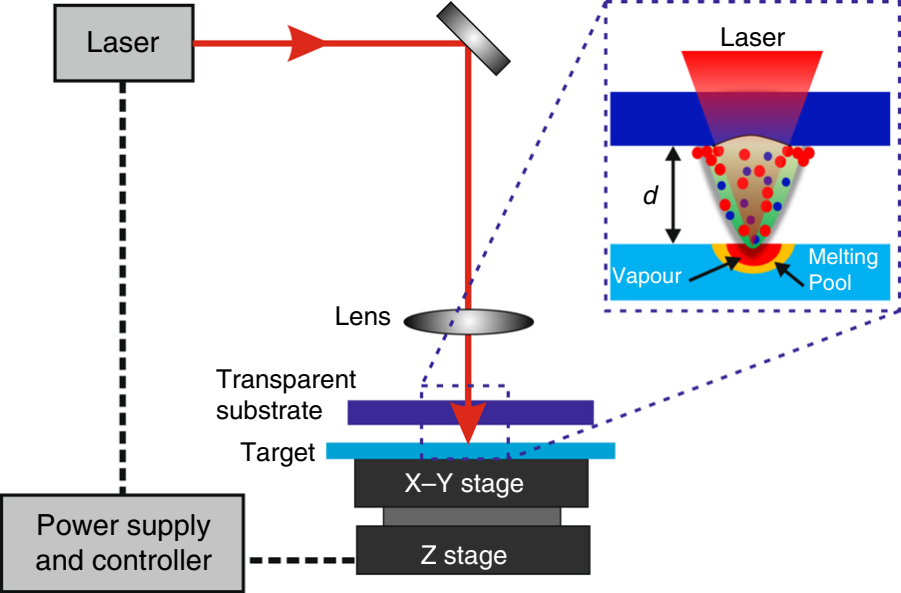
Schematic of LIPAA setup. The setup mainly consists of a laser, a transparent substrate and a target. The laser beam passes through the the transparent substrate and irradiates on the target which is located behind the transparent substrate. The target is highly absorptive to the laser energy and laser action on the target surface can induce an amount of plasma. The plasma fly towards the rear surface of the substrate at a high speed and assist laser ablation. *d* is the distance between the transparent substrate and the substrate

Most laser machining processes are realized via laser-induced ablation, where the laser energy interacts with the substrate and leads to the removal of substrate materials. Therefore, it is crucial that laser energy is absorbed adequately by the substrate for effective laser ablation. However, it is difficult to meet this requirement for transparent materials due to their high transparency. To overcome this challenge, Zhang et al.^32^ proposed a LIPAA process, where plasmas generated from the interaction between an incident laser and a metal target are utilized to enhance the laser absorption of quartz material. This technique is applicable to all transparent materials. Figure 1 shows a schematic of the LIPAA setup, which consists of a laser, a substrate, and a target. The substrate must be transparent to the laser wavelength. The laser beam, at a laser fluence below the substrate ablation threshold, can pass through the transparent substrate, and then irradiates on the target which is located behind the transparent substrate at a distance of *d*. As the target is highly absorptive to laser energy, laser action on the target surface can induce a high-temperature and high-pressure plasma, which flies forward the rear surface of the substrate at a high speed. Strong interactions among laser, target plasma, and substrate, and target materials deposition take place at the rear surface and lead to absorption enhancement to the incident laser energy. As a result, although the laser fluence is lower than the ablation threshold of the transparent hard substrate, localized laser ablation can be achieved at the rear surface with the assistance of the laser-induced plasmas. The target-substrate distance *d* is a critical factor that has great impacts on the processing parameters, such as ablation rate and ablation width. With the increase of the distance *d*, the target plasma takes a longer time to reach the substrate with lower kinetic energy. Generally, the ablation rate decreases as the *d* increases due to the weaker plasma assistance^32,43,44^. The ablation width may decreases or increases depending on the laser fluence and processing parameters^43–46^. For actual applications, the distance *d* is typically from 0 to a few millimeters depending on the laser fluence, pulse width, and requirement of ablation resolution. In addition to solid targets, laser-induced plasma in liquids can also be used to process transparent materials^47^. Different from LIPAA using solid targets, the transparency of liquid materials allows laser processing from the front surface.

There are unique advantages to the LIPAA in the micromachining of transparent hard materials. First, due to the absorption enhancement, this technique is capable of ablating transparent materials effectively using conventional visible or IR lasers. Second, the use of laser-induced plasma causes a reduction of ablation threshold^45^, allowing us to use relatively low laser energy for precision engineering. Therefore, LIPAA can give rise to the cost reduction of equipment and operation and reduce the risk of micro-cracks formation owing to less heat accumulation. Third, the laser process efficiency and productivity can be greatly improved due to the low ablation threshold and the assistance of laser-induced plasma. A comparative study shows that the processing time of LIPAA is reduced up to 84% compared with the direct laser ablation^47^.

LIPAA has been extensively studied for microstructures, micro-patterns, and optical devices fabrication on a variety of transparent substrates, including glasses, quartz, and sapphire. Metals and semiconductors, such as copper (Cu)^26^, silver (Ag)^48,49^, aluminum (Al)^26,50^, sapphire powder^51^, silicon (Si)^45,52^ and distilled water^47,53^ are used as target materials. The most common light sources used in LIPAA are nanosecond pulse lasers with wavelengths ranging from UV to IR. Femtosecond laser LIPAA is also reported for micro/nano-processing of transparent materials^45,54^. The use of femtosecond laser delivers more advantages and is capable to achieve even higher quality and lower roughness microstructures fabrication. Table 1 provides a summary of LIPAA studies for different transparent hard substrates using different lasers and targets. The details of these studies are discussed in the following sections.

**.Table 1:** Summary of LIPAA studies on different transparent hard materials

Substrates	Laser wavelength and pulse duration	Targets	Applications	References
Fused quartz	266 nm, 6 ns	Stainless steel	Micro-grating structures	^32^
	248 nm, 34 ns	Stainless steel 304	Micro-grating fabrication	^61^
	532 nm, 6 ns	Ag	Micro-grating structures and micro-hole drilling	^48^
	266 nm, 532 nm, and 1064 nm, 6 ns	Ag	Surface structuring and channel drilling	^49^
	1064 nm, 5 ns	Si	Ablation	^52^
Pyrex glass	266 nm, 532 nm, and 1064 nm, 6 ns	Ag	Micro-grating structures and channel drilling	^49^
	532 nm, 6 ns	Ag	Micro-grating structures and micro-hole drilling	^48^
Glass substrates	532 nm	Al, Si, SiC, Cu	Surface printing and marking	^50^
	248 nm, 34 ns	Cu, Al	Color marking and metal film deposition	^26^
Silicate glass	775 nm, 180 fs; 532 nm, 500 ps; 532 nm, 9 ns	Al, Cu, Pb_3_O_4_, Cr_2_O_3_, and Cu(C_32_H_15_ClN_8_)	Color marking	^65^
Soda-lime glass	1064 nm, 100 ns	Graphite	Electrofluidic devices	^66^
	1064 nm, 100 ns	AlSiC, Al alloy	Microfluidic devices	^46^
Matsunami glass slides	248 nm, 23 ns; 532 nm, 6 ns	Si, Cu, and Ag	Micro-patterning and color marking	^60^
Sapphire	1064 nm, 150 ns	Ni-coated steel	LED wafer scribing	^68^
	800 nm, 50 fs	Si	Microstructures fabrication	^45^
	800 nm, 50 fs	Cu	Optical devices fabrication	^54^
	1064 nm, 1–5 ns	Cu	Ablation and Metallization	^67^
CaF_2_	248 nm, 20 ns	Brass	Microstructuring	^62^
Alumina ceramic	532 nm, 8 ps	Distilled water	Fabrication of micro-channels	^47,53^
Diamond	532 nm, 8 ps	Distilled water	Fabrication of micro-channels	^183^

### Dynamic process and physical mechanisms of LIPAA

LIPAA is a highly dynamic process. In a time scale of nanoseconds, there are laser-target interaction, plasma generation, ablation of a target, target material deposition, and complicated interactions among plasma, laser, and substrate. LIPAA process starts with the absorption of photons by the target material, followed by the heating and photoionization of the irradiated area by the laser beam. Subsequently, the laser-induced plasma is released from the target together with solid particles, vapors, and liquid drops. The physical process behind the laser-induced plasma varies from material to material. Most of LIPAA processes use metal materials as the target. In metal, there are a large number of free electrons available. The laser energy is absorbed by free electrons through the inverse bremsstrahlung mechanism. As free electrons are excited, the collision rate increases, and the energy is transferred from the excited electrons to lattice phonons. When the lattice temperature is high enough, melting, vaporization, and plasma expansion occur. The plasma may be generated through several different mechanisms depending on laser parameters. For nanosecond laser irradiation with intensity < 10^8^ W/cm^2^, the dominant mechanism of the laser-induced plasma is thermal vaporization, whereas for femtosecond laser with intensity >10^13^ W/cm^2^, the main laser-induced plasma mechanism is non-thermal Coulomb’s explosion^55^. For laser intensity between 10^8^ and 10^13^ W/cm^2^, both two mechanisms contribute to the laser-induced plasma. When dielectric targets are used, the laser energy first causes ionization and liberates electrons in the materials. Taking distilled water as an example, for nanosecond laser pulses, the generation of free electrons is initiated by collisional or multiphoton ionization. Once free electrons are available, avalanche ionization dominates the ionization process. For ultrashort laser pulses (picosecond and femtosecond), multiphoton ionization plays the dominant role^56^. The generated plasma further interacts with the incoming laser and results in the inverse bremsstrahlung absorption which causes the isothermal expansion of the plasma. The expansion plasma reaches and interacts with the rear surface of the substrate.

**Fig. 2:**
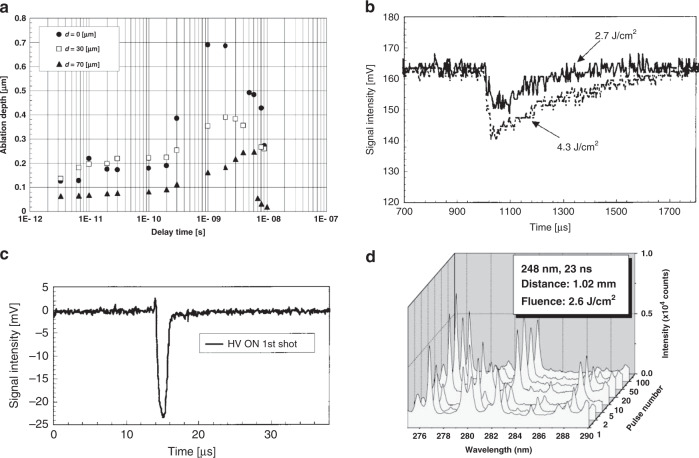
Studies of the dynamic process of LIPAA. **a** Dependence of ablation depth on delay time between the 1st and the 2nd pulses for target-to-substrate distances of 0, 30, and 70 μm^57^. **b** Transient absorption enhancement of the substrate due to the laser-induced plasma at laser fluences of 2.7 and 4.3 J/cm^2^. The target-to-substrate distance is 12 mm^58^. **c** Plasma-conductivity signal observed in fused silica irradiated with an F_2_ laser, indicating that electron–hole pairs are formed^58^. **d** Optical emission spectra during the KrF excimer LIPAA of fused quartz at a target-to-substrate distance of 1.02 mm and a laser fluence of 2.6 J/cm^2^. At the first pulse, the quartz spectral line intensity is almost zero, indicating there is no laser ablation of the substrate^60^. Figure **a** reproduced with permission from ref. ^57^,2005 Elsevier B.V. All rights reserved; **b**, **c** from ref. ^66^, © 2006 American Institute of Physics; **d** from ref. ^58^, © 2002 Elsevier Science B.V. All rights reserved

Transient phenomena of the interaction between laser-induced plasma and transparent substrate were studied using double-pulse femtosecond laser irradiation^57^. A femtosecond laser beam (775 nm, 180 fs, 1 kHz) is divided into two beams. The first beam generates plasma from a Cu target, and the other one is used for substrate ablation. The study shows that the ablation depth of the second beam is highly dependent on the time delay between the two pulses at a fixed target-to-substrate distance, and the moment of maximum ablation depth varies from the target-to-substrate distance. As shown in Fig. 2a, the ablation depth reaches the maxima at the delay time of 1, 3, and 6 ns for different target-substrate distances of *d* = 0, 30, and 70 μm, respectively. This phenomenon is attributed to the influence of laser-induced plasma. The plasma interacts with the substrate at the rear surface and enables a transient absorption enhancement, which has been verified by transient absorption measurement^58^ shown in Fig. 2b. The absorption enhancement takes place with a delay after the laser pulse strikes the target^57,59^. When the time delay of the second pulse is tuned to synchronize with the moment of the absorption enhancement, the maximum ablation depth is observed. The mechanism behind the transient absorption enhancement was also investigated^58^. High energy ions in laser-induced plasma play the most important role. Plasma-conductivity measurement (Fig. 2c) reveals that ions in the plasma with energy above the critical kinetic energy can excite electrons and generate excited states inside the transparent substrate, resulting in a transient strong absorption of the incident laser and a significant increase of laser ablation.

**Fig. 3:**
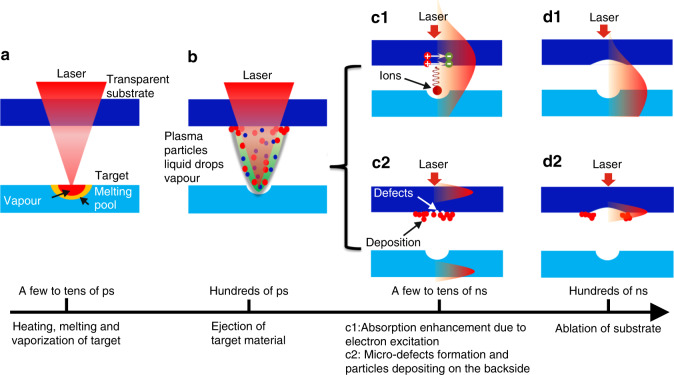
Schematic illustration of LIPAA process. **a** Heating effect of the laser irradiation causes localized melting and vaporization. **b** The laser-induced melting and vaporization lead to the ejection of target materials. **c1**, **d1** Case1: there is an overlap between the later part of the pulse and the time for absorption enhancement originated from the laser-induced plasma. Laser ablation occurs due to strong absorption enhancement at the rear surface. **c2**, **d2** Case 2: there is no overlap between the laser pulse and the absorption enhancement. The absorption enhancement from the micro-defects and deposition results in the ablation by subsequent pulses

Figure 3 summarizes the dynamic process of LIPAA. When a laser pulse strikes the target, the pulse energy is absorbed and therefore heats the target surface. Subsequently, the heating effect causes localized melting and vaporization. The induced pressure wave leads to the ejection of a small volume of materials from the target surface in the form of plasma, particles, vapors, and liquid drops, which is known as laser ablation. High-speed ions in the ejection then collide with the rear surface and interact with the substrate. When the ion kinetic energy is above 10 eV, the interaction can induce electron-hole pairs on the substrate and causes the absorption enhancement. A few hundred picoseconds are needed for the plasma generation and electron excitation, and it also takes a few nanoseconds, typically, between zero to tens of nanoseconds depending on the target-substrate distance, for the ions to reach the substrate at a speed around 10^4^ m/s^57,59^. Thus, the ablation mechanism of LIPAA can be divided into two basic types depending on whether the laser interacts with the plasma. First, when a long pulse laser is used (typically, >10 ns) and the target-substrate distance is small (typically, <200 μm), the pulse duration is larger than the time of plasma generation and plasma flight. There is an overlap between the later part of the pulse and the time for absorption enhancement originated from the laser-induced plasma. The laser energy is strongly absorbed by the rear surface and ablation occurs there (Fig. 3c1, d1). Second, if the pulse duration is short or the target-substrate distance is large, e.g., *t* = 23 ns, *d* = 1.02 mm in reference^60^, the arrival of the laser-induced plasma at the substrate backside surface is after the laser pulse terminates. There is no overlap between the laser pulse and the absorption enhancement. The first pulse passes through the substrate without ablation due to inadequate laser energy absorption, as shown in Fig. 2d. In this case, the study on optical emission spectra shows that laser ablation still can be achieved by the subsequent pulses^60^. The plasma can generate micro-defects and deposit target materials on the substrate backside, which serves as new laser energy absorption centers. The absorption enhancement from the micro-defects and deposition eventually results in the backside ablation of the transparent substrate.

### Nanosecond laser LIPAA and its applications

The most commonly used lasers for LIPAA are nanosecond lasers attributed to their remarkable advantages like cost-effectiveness, low maintenance requirement, and high reliability over picosecond and femtosecond lasers. However, the long pulse duration leads to the continuous heating of the target material. The laser energy spreads by heat conduction to a large area outside the laser spot, causing the target to boil and evaporate. Boiling and evaporation of the target material lead to the production of large particles and even bulky target materials, which deposit on the rear surface of the transparent substrate. As a result, the LIPAA processed surface is much rough due to the non-uniform deposition. Another shortcoming is that it is difficult for the nanosecond laser LIPAA to fabricate deep structures. This is because the processed area becomes rough and opaque once a layer is removed from the surface. Thus, the laser transmittance drops, and the LIPPA effect becomes weaker. A variety of nanosecond laser sources, such as excimer, solid-state, fiber lasers, and their frequency-converted lasers, with wavelength range from deep UV to IR, have been applied to the LIPAA process. There are various practical applications of this high-quality LIPAA method for microstructuring, surface micro-patterning and marking, micro-hole drilling, and precise cutting of transparent hard materials.

**Fig. 4:**
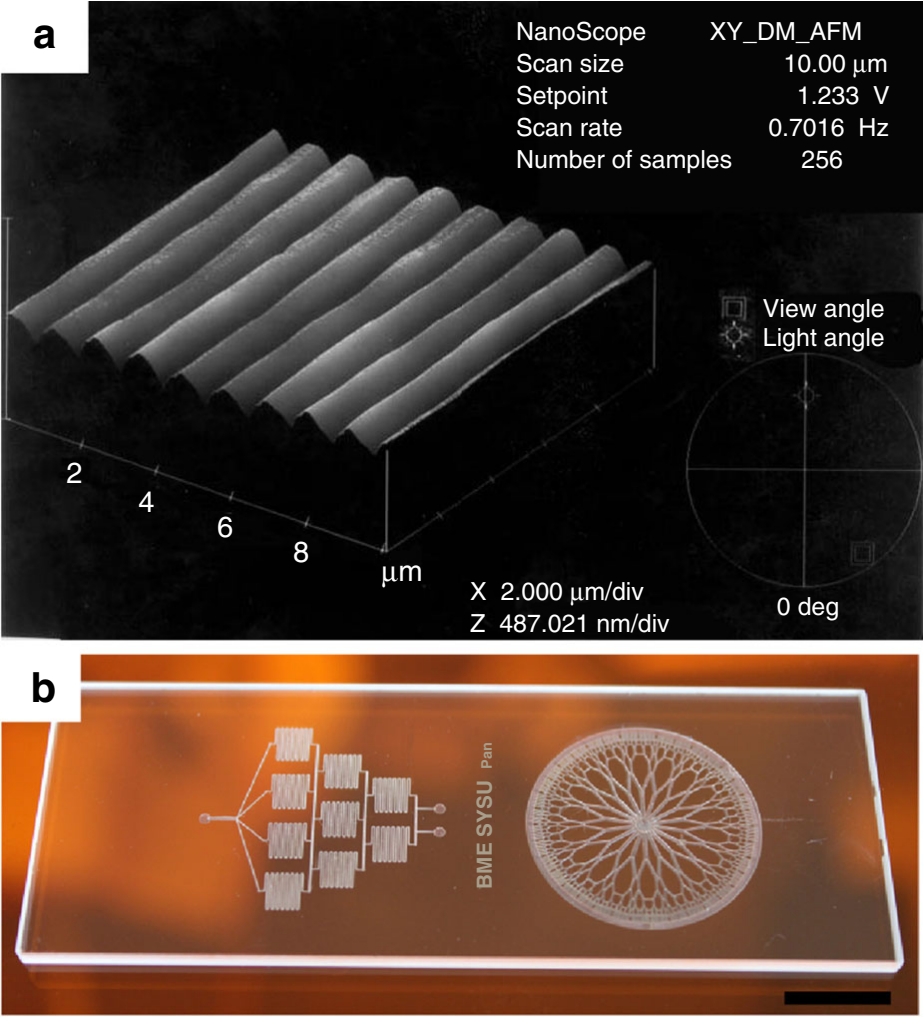
Microstructuring of transparent substrates by LIPAA. **a** AFM images of a grating fabricated on fused silica using a KrF excimer laser and a phase mask^26^. The grating shows a period of 1.06 μm and a clean and well-defined structure without any severe damage. **b** Two microfluidic device patterns fabricated on soda-lime glass substrate by LIPAA^46^, scale bar: 150 μm. Figure **a** reproduced with permission from ref. ^26^, ©Springer-Verlag 2003, and **b** reproduced with permission from ref. ^46^, ©2016 Elsevier B.V. All rights reserved

One of the primary applications of LIPAA is laser microstructuring and patterning by selectively ablating the rear surface of substrates. It was first demonstrated on fused quartz substrate by the fourth harmonic of an Nd^+^: YAG (266 nm) laser^32^. The substrate was easily etched by the laser beam with the assistance of the laser-induced plasma, although it is transparent to the laser wavelength. A fine grating structure with a line spacing of 20 μm without any severe damage was fabricated. Then this technique was extended to other transparent substrates like silicate glass, soda-lime glass, and calcium fluoride (CaF_2_), and extensively studied for different microstructures and patterns fabrication^46,48,50,61^. Microstructuring of spiral, relief “I” shape and 3D pyramid structure on glasses have been demonstrated by adjusting laser scanning mode using the second harmonic of an Nd^+^: YAG laser (532 nm)^26,50^. High-quality micro-grating with a 1.06 μm period and a 300 nm depth have been fabricated on quartz substrate using a phase mask through precise control of laser parameters^26^, as shown in Fig. 4a. The feasibility of micro-channel fabrication on soda-lime glass was also demonstrated by taking advantage of LIPAA and chemical corrosion^46^, which provides an effective method to fabricate glass microfluidic devices. In many applications, it is important to control the feature sizes of the microstructures. A number of laser processing parameters, such as target-substrate distance, laser fluence, scanning speed and pulse number affect the LIPAA process. Studies on the parameters’ influence show that ablation rate, structure depth, and width are highly dependent on these processing parameters^32,44,46,60,62^. Thus, the microstructure feature sizes can be precisely controlled by optimizing the processing parameters for high-quality and precise microstructures fabrication.

**Fig. 5:**
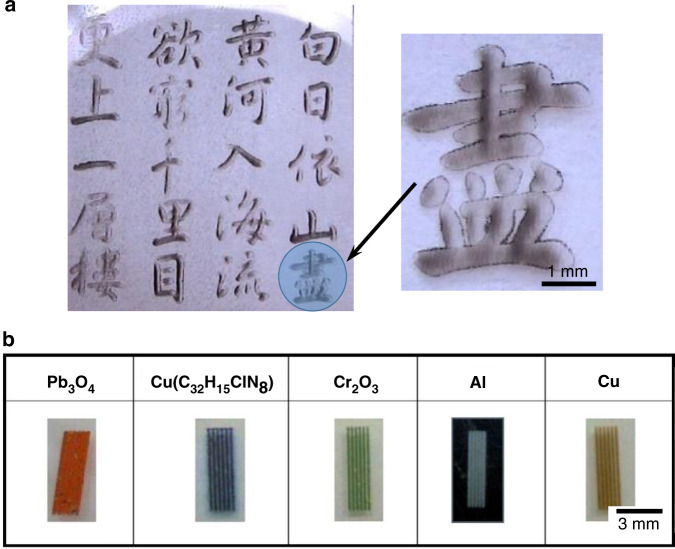
Laser printing and color marking of transparent materials by LIPAA. **a** Microscopy images of a Chinese poem printing on glass substrate^50^. **b** Laser color marking on silicate glass substrates using different targets of Pb_3_O_4_ for red, [Cu(C_32_H_15_ClN_8_)] for blue, Cr_2_O_3_ for green, Al for white, and Cu for brown^65^. Figure **a** reproduced with permission from ref. ^50^, © (2001) Copyright Society of Photo-Optical Instrumentation Engineers (SPIE), and **b** from ref. ^56^, © 2007 IOP Publishing Ltd.

By means of computer-aided design to control the laser scanning patterns, the LIPAA process can serve as a flexible printer, printing any patterns on transparent substrate surfaces. Laser printing of texts, characters, even pictures on glasses has been demonstrated^26,44,50,63,64^, where the patterns are produced by either the ablation of the substrate or deposition of the target materials on the substrate backside surface. Laser printing of a Chinese poem on a glass substrate by LIPAA has been demonstrated, as shown in Fig. 5a^50^. The LIPAA processed areas exhibit an opaque pattern and produce excellent contrast on the transparent substrate. When colorful targets are used, LIPAA offers an alternative process for high-quality and high visibility color marking on a transparent substrate. Black, white and red color drawings were also printed on glass substrates using different target materials, including SiC, Al, and Cu, respectively^50^. The effect of target-substrate distance on color tones was studied, where it is found that the color tones of the LIPAA induced marking can be controlled by adjusting the target-substrate distance. By using colorful metallic compounds as targets, such as Pb_3_O_4_, Cr_2_O_3_, and Cu(C_32_H_15_ClN_8_), laser marking of red, green, and blue colors on silicate glass substrates have also been demonstrated^65^, as shown in Fig. 5b. Further studies of the parameters’ influence suggest that strong adhesion can be achieved by using long-duration pulses at a short target-substrate distance.

**Fig. 6:**
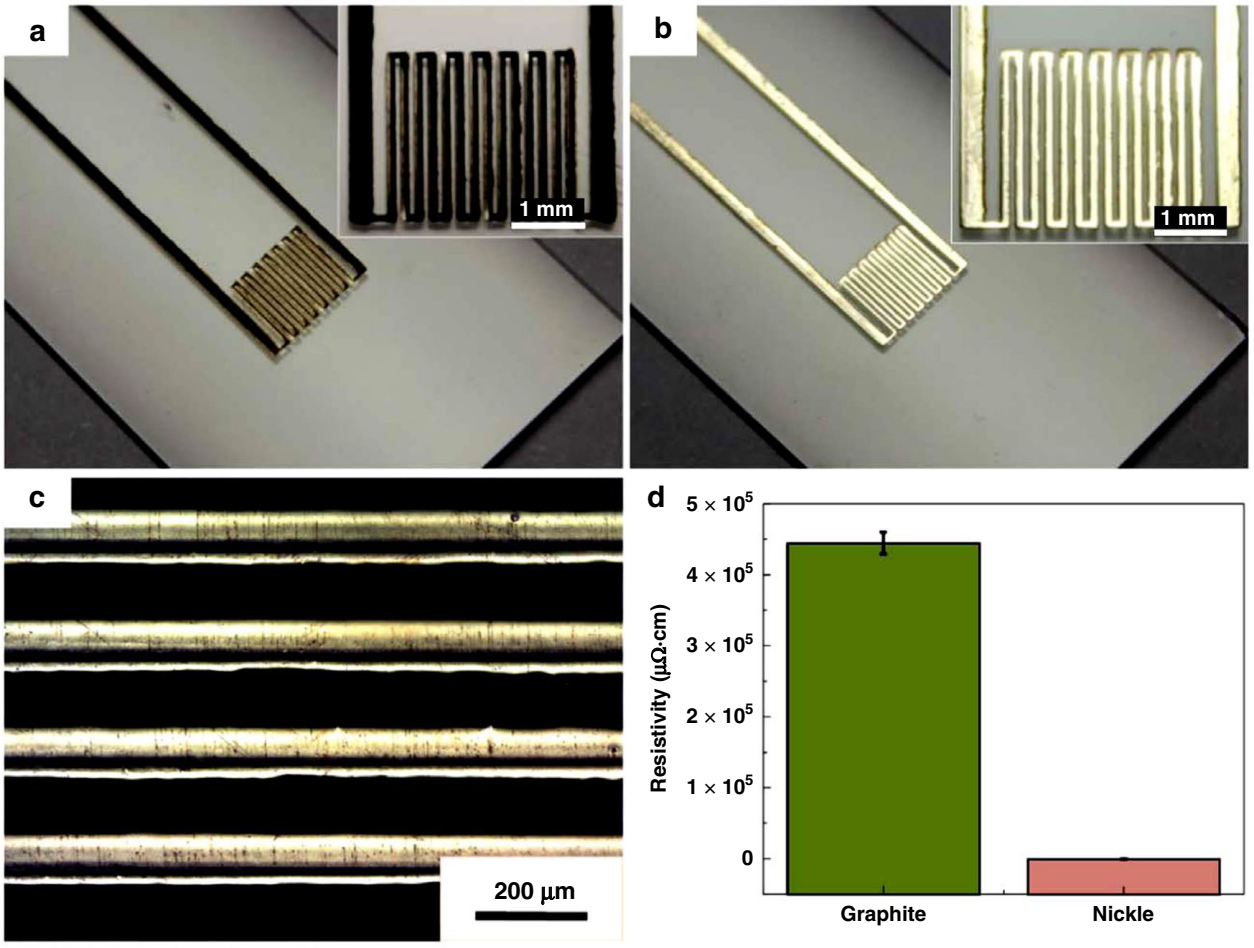
Demonstration of micro-electronics devices fabricated by combining LIPAA and electroplating^66^. **a** Graphite patterns fabricated by LIPAA, and **b**, **c** Ni patterns coated by electroplating. **d** Resistivity of the graphite and Ni patterns. Reproduced with permission from ref. ^66^, © 2017 Elsevier B.V. All rights reserved

Careful control of the LIPAA processing parameters can enable thin metal film deposition, which offers a promising technique for metalized structures formation on transparent substrates. Hong et al. have demonstrated the feasibility of this method to fabricate electronic circuits on glass substrates using metal target materials^26,50^. The circuit was then used as an electrode to perform electroplating experiments, and it can be converted into copper patterns by electroplating coating using CuSO_4_ solution. The combination of the LIPAA process and electroplating is capable of fabricating micro-electronics devices on transparent substrates. An example is shown in Fig. 6^66^, where a Ni-patterned electrofluidic device is fabricated by LIPAA combined with electroplating. In this study, electroplating can significantly enhance the conductivity and adhesive strength of the electrode. The conductivity of the metal film generated by LIPAA has been systematically investigated^44,67^. The resistivity is highly dependent on the LIPAA processing parameters, such as scanning speed, target-substrate distance, repetition rate, and laser fluence, which means that the resistivity can be precisely tuned by adjusting the processing parameters.

LIPAA can be also applied for transparent substrate scribing and cutting. Sapphire is widely utilized in various industries, including LED manufacturing and smartphone fabrication. UV nanosecond lasers (355 or 266 nm) and ultrashort lasers are often used to cut or scribe sapphire substrates. Owing to the unique effect of the laser-induced plasma, LIPAA makes it possible to use conventional IR or visible nanosecond pulse lasers for sapphire cutting and machining^68^. These widely used lasers in the industry certainly can enhance advanced manufacturing of next-generation functional devices on transparent hard materials.

### Femtosecond laser LIPAA and its applications

In general, femtosecond lasers have extremely high peak power and short pulse duration, which offers the advantage of high precision processing with minimized heat-affected zones^28,38^. For the case of femtosecond laser LIPAA, as discussed above, micro-defects and particle deposition produced on the transparent substrate backside surface play a dominant role in the absorption enhancement and laser ablation. The target material is able to be directly evaporated into vapor from the solid-state owing to the extremely high pulse peak power. Such solid-to-vapor phase transition involves the creation of high-energy plasma species following a rapid expansion. This rapid process can produce a much stronger plasma with higher kinetic energy, smaller size particles, and fewer molten materials than nanosecond lasers^69^. Therefore, the use of femtosecond pulses can produce higher quality, more uniform micro-defects, and particles’ deposition with less thermal damage to the substrates. Studies have confirmed that the femtosecond laser LIPAA offers significant advantages in terms of debris, cracks, and surface roughness^45,54^. There are two main shortcomings of the femtosecond laser LIPAA: (1) High cost of laser equipment and maintenance compared to nanosecond lasers. (2) The high surface quality is achieved at the cost of ablation rate. Therefore, femtosecond LIPAA is suitable for the fabrication of precision microstructures on transparent substrates.

**Fig. 7:**
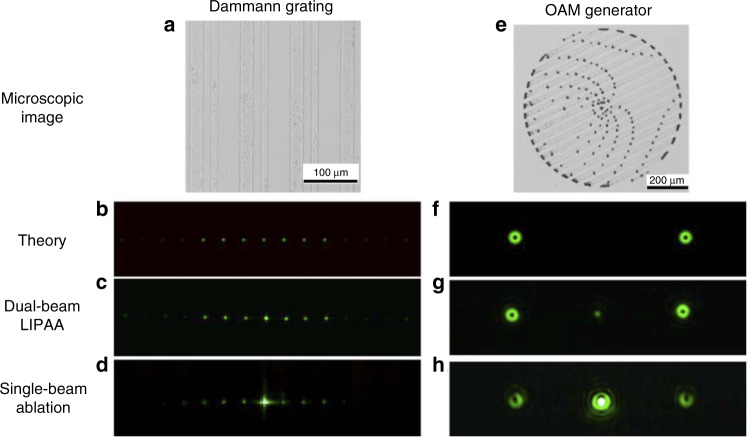
Optical components fabricated by dual femtosecond laser beam LIPAA. **a**, **e** Microscopic images of a Dammann Grating and an OAM generator fabricated on sapphire. **b**, **f** Theoretical simulation of the diffraction pattern of the Dammann Grating and OAM generator. **c**, **d** Measured diffraction patterns of the Dammann Grating fabricated by dual-beam LIPAA and single-beam femtosecond laser ablation, respectively. **g**, **h** Measured diffraction patterns of the OAM generator fabricated by dual-beam LIPAA and single-beam femtosecond laser ablation, respectively^54^. Reproduced with permission from ref. ^54^, © 2020 Optical Society of America

An example of femtosecond laser LIPAA is demonstrated in work^54^, where dual femtosecond laser beams were used to overcome the problem of high roughness of direct laser ablation. In this design, one laser beam is focused onto a Cu target to generate plasmas and nano-particles. The other beam is used for the ablation of the transparent hard substrate. Comparative studies show that such femtosecond laser LIPAA not only increases ablation rate but also significantly reduces the processing roughness. The ablation rate of the dual-beam femtosecond laser LIPAA can be increased by 47.5% than that using single-beam. In terms of processing roughness, the surface roughness generated by nanosecond laser LIPAA is around 100 nm^62^, while the femtosecond laser LIPAA can realize a much lower roughness, down to 18.1 nm. The low roughness is able to meet the roughness requirement of optical components for visible light applications (Ra < *λ*/20). Fabrication of diffractive optical elements has been demonstrated on sapphire substrates by the femtosecond laser LIPAA, as shown in Fig. 7. The fabricated Dammann grating and orbital angular momentum (OAM) generator exhibit well-defined geometries and excellent diffraction performance. The modulation efficiency of the Dammann grating and the OAM generator were greatly improved from 41.5% and 39.1% (single-beam ablation) to 85.7% and 92.3%, respectively, by the dual-beam LIPAA^54^.

**Fig. 8:**
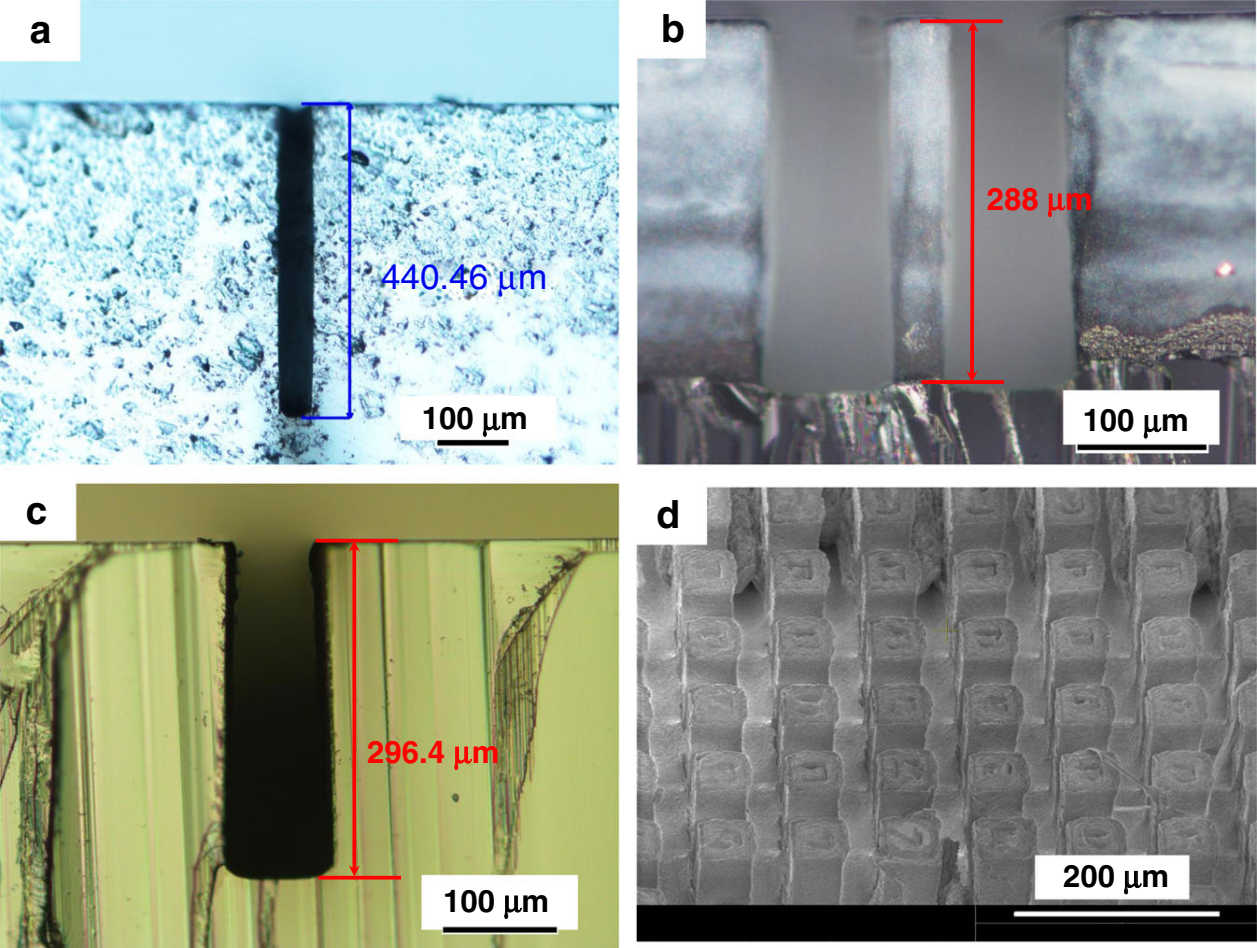
Microstructures fabricated in sapphire by femtosecond laser LIPAA^45^. **a** Taper-free, high-aspect-ratio (>10) deep micro-channel. **b** Single micro-column. **c** Trapezoid micro-channel. **d** Micro-column array. Reproduced with permission from ref. ^45^ © 2020 Elsevier Ltd. All rights reserved

Another example of the femtosecond LIPAA was demonstrated in Fig. 8^45^. It is found that the femtosecond LIPAA is able to overcome the problem of micro-crack formation on the sapphire substrate during the laser processing owing to low thermal conductivity and the high linear thermal expansion coefficient of sapphire. Since laser fluence has a great effect on the ablation rate, the ablation rate can be precisely controlled to match the substrate movement speed, which allows the laser ablation of sapphire at a critical laser fluence slightly above the ablation threshold. Such critical laser fluence can effectively avoid crack formation. Crack-free and taper-free microstructures with an aspect ratios greater than 10 were fabricated on sapphire substrates, as shown in Fig. 8a. This method is also used to fabricate a variety of other functional microstructures, which are difficult to be achieved by other methods due to the hardness and fragility of the substrate (Fig. 8b–d). The structures’ surfaces exhibit no obvious cracks and debris, indicating that the quality is much higher than that of micro-channels and holes fabricated by nanosecond laser LIPAA^48,49^.

## Laser-induced backside wet etching

### Characteristics and methodology of LIBWE

LIBWE is another hybrid laser micromachining technique, which takes advantage of liquid to enhance the absorption of transparent substrates. In such a technique, a highly absorptive liquid is in contact with one side of the transparent substrate and a laser source irradiates from the other side. The laser beam is focused onto the solid-liquid interface after passing through the substrate. The temperature at the interface increases rapidly due to the absorption enhancement of the absorptive liquid, which leads to a significant reduction of the ablation threshold. The effect of LIBWE on the ablation threshold has been studied in works^70,71^. The threshold fluence of quartz is reduced to 0.5 J/cm^2^ using a solution of pyrene in acetone (concentration, 0.4 M/L) from 20 J/cm^2^ under direct laser irradiation^70^. A study on water-assisted femtosecond laser processing also shows that the ablation threshold of fused silica is reduced from 2.22 to 1.02 J/cm^2^ with the assistance of distilled water^71^. Removal of the substrate material may occur through several different processes including material softening, transient high pressure, thermoelastic stress, and shock wave generation. In addition to absorption enhancement, the liquid absorber also plays an important role in heat dissipation and taking away debris produced during the laser etching. LIBWE is first demonstrated by H. Niino’s group for the micromachining of fused silica using an organic liquid, pyrene/acetone solution^33^. It has been extensively investigated on various substrates, such as fused silica, soda-lime glass^72,73^, quartz crystal^70^, sapphire^74–76^, CaF_2_^77^, and MgF_2_^77^, using different liquid absorbers, such as hydrocarbon solvents, organic solutions, metal salt, liquid metal, inorganic compounds solved in organic solvents, and even water^71,78,79^. Table 2 summarizes the studies of LIBWE on different materials and makes a comparison in terms of the laser wavelength, pulse duration, absorptive liquid, and applications.

**Table 2:** Summary of LIBWE studies on different transparent hard materials

Substrate materials	Laser wavelength and pulse duration	Absorptive liquid	Fabricated structures and applications	References
Fused silica	248 nm, 30 ns	Pyrene/Acetone solution	Grating microstructures	^33^
	351 nm, 30 ns	Pyrene in acetone, tetrachloroethylene, or toluene	Laser etching	^72^
	248 nm, 30 ns	Pyrene/Acetone solution	Micro-trench	^83^
	248 nm, 25 ns	Mercury	Laser etching of groove	^92^
	248 nm, 20 ns	Liquid gallium	Laser etching of micro-pits	^93,184^
	266 nm and 355 nm, 10 ps	Toluene	Micro-grooves	^80^
	248 nm, 30 ns	The naphthalene-1,3,6-trisulfonic acid trisodium salt	Periodic line and grid patterns	^96^
	308 nm, 20 ns	Pyrene/Acetone solution	Micro-etching of grating structures	^99^
Silica glass	800 nm, 120 fs	Distilled water	3D micro-holes	^78^
	800 nm, 40 fs	Distilled water	3D mciro-channels	^100^
	800 nm, 120 fs	Distilled water	3D microfluidic chips	^98^
Foturan glass	1045 nm, 457 fs	Distilled water	Glass microfluidic structures	^81^
Alumino-borosilicate glass	n.s., 250 fs	Water	Opto-mechanical modulators	^185^
Soda-lime glass slide	1064 nm, n.s.	CuSO_4_	Laser etching and cutting	^73^
Quartz	355 nm, 30 ns	Toluene	Micro-channel fabrication	^101^
	248 nm and 308 nm, 30 ns	Pyrene/acetone solution	Fresnel lens	^70^
	308 nm, 30 ns and 200 ns	Pyrene in tetrahydrofuran	Micro-lens array	^84^
Sapphire	1064 nm, 100 ns	CuSO_4_	Laser etching and cutting	^74^
	266 nm, 150 ps	Chlorobenzene	Periodic gratings	^82^
	248 nm, 30 ns	Solution of pyrene/acetone or neat toluene	Laser patterning, grating structures	^75^
CaF_2_	248 nm, 30 ns	Pyrene in acetone, tetrachloroethylene or cyclo-hexane	Laser etching	^77^

*n.s.* not specified

Table 2 indicates that most studies of LIBWE use nanosecond lasers. Because it is difficult for nanosecond laser to directly process transparent hard materials, additional assistance, i.e., absorptive liquid is introduced to enhance the laser energy absorption and reduce the threshold fluence. In addition, lower cost of equipment and maintenance, higher reliability of the nanosecond lasers compared to ultrafast lasers (picosecond or femtosecond laser) are also important factors for the choice of the laser source, which is similar to the LIPAA process. Some of the studies used ultrafast lasers under the consideration of low heat affected zone, high resolution, and high surface quality^80–82^. Table 2 also shows that the laser wavelength used in the LIBWE process ranges from VUV to near IR.

**Fig. 9:**
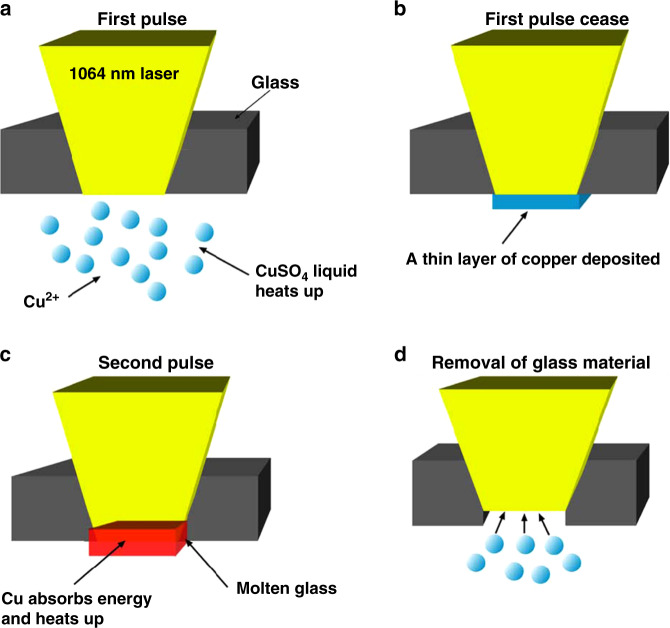
Proposed mechanism of LIBWE using 1064 nm laser and CuSO_4_ solution as the liquid absorber^73^. **a** Laser irradiates from the top. **b** Copper deposition on the backside of the glass. **c** The deposited copper absorbs the laser energy and heats up the immediate glass region. **d** Removal of the molten substrate. Reproduce with permission from ref. ^74^, © Springer-Verlag 2008

Several physical mechanisms have been suggested to explain the LIBWE process depending on the liquid absorber used. The authors’ research group conducted an experimental study on the LIBWE by using aqueous copper sulfate (CuSO_4_) solution as an absorber^73^. A two-step model, as shown in Fig. 9, was proposed to describe the etching mechanism. The first step is the deposition of copper on the glass surface due to a photochemical process upon laser irradiation. It is followed by the second step of absorption of the laser energy by the deposited copper, resulting in melting and removal of the glass materials. Studies on cavitation bubble dynamics show that bubbles are favorable to the decomposition of CuSO_4_, the material removal, and supplying fresh solution to the substrate surface for further etching progress^74,83^. For the case of hydrocarbon solvents or solutions, the physical process has been discussed^82,84–89^. At the liquid–solid interface, laser energy is strongly absorbed by a thin liquid layer that is contacting with the transparent substrate surface. The liquid temperature rises dramatically and heats the nearby solid surface, which causes a localized softening even melting of the substrate material when the temperature is high enough. Meanwhile, high temperature and high-pressure bubbles are generated in the boiling liquid due to the superheated liquid. The bubbles expand rapidly and induce strong mechanical effects at the solid-liquid interface, resulting in the removal of the softened substrate materials from the backside surface. In addition, the laser-induced decomposition of the organic molecules and the carbon deposition on the substrate surface also affect the etching process by changing the physical and chemical properties of the solid-liquid interface^86,90,91^. For the case of liquid metals like gallium and mercury^92,93^, the LIBWE is a thermal process comprising of laser heating of liquid, heat transfer to the transparent substrate, and removal of molten and/or evaporated substrate, which is similar to using hydrocarbon solvents or solutions. However, it does not involve the decomposition process due to the pure liquid metals.

### Optimization of LIBWE: toward high etching rate and high surface quality

As a hybrid laser processing, LIBWE makes use of highly absorptive liquid to enhance absorption, allowing the well-defined microstructuring of transparent materials at a relatively low laser fluence using a simple setup. For practical applications, it is important to precisely control the etching rate and surface quality. A large number of factors and parameters are involved in the LIBWE process including liquid properties, solution concentration, scanning speed, and laser fluence^94^. Careful optimization of processing parameters leads to the precision laser engineering of transparent hard materials toward a high etching rate and high surface quality. First, the property of the liquid absorber is a critical factor that greatly affects laser energy absorption and material removal. The etching rate and surface quality vary from different liquids. Usually, the etch rate of fused silica ranges from a few nm to tens of nm per pulse using pyrene-doped hydrocarbon solution^72,88^. Niino et al.^90^ demonstrated that the etch rate of pure toluene is about 30% higher than that obtained in a pyrene-doped acetone solution at a concentration of 0.4 M at the same condition. Higher etch rates of LIBWE have been observed using liquid metal as the absorber^92,93^. Etch rate above 600 nm/pulse is achieved, which exceeds the etch rate using organic absorbers by more than 10 to 100 times, meanwhile maintaining a smooth and high-quality surface. Another factor affecting the etch rate is the solution concentration. Higher solution concentration has a larger absorption coefficient, leading to a larger etching rate^33,77^. The etch rate also has a strong dependence on laser fluence^70,80,90,93^ and grows almost linearly with laser fluence. It can be tuned over a wide range from a few ns/pulse to above 600 nm/pulse by adjusting the laser fluence from 0.5 to 8 J/cm^2^ ^93^. Comparative study of LIBWE using different wavelengths shows that short-wavelength has a higher etch rate^70,80^. The etch rate with *λ* = 266 nm is approximately about 5 times higher than with *λ* = 355 nm at the same condition, which can be attributed to the higher absorption coefficient of the liquid at a shorter wavelength.

On the other hand, high-quality laser processing often requires a relatively low etch rate. As discussed above, etch rate varies greatly with solution concentration and laser fluence. Low etch rate and nanometer-scaled depth control can be achieved by applying a low laser fluence or a low solution concentration. The etch rate can be reduced from 25 to 5 nm/pulse when laser fluence decreases from 1300 to 400 J/cm^2^ ^33^, and it can be further reduced to below 1 nm/pulse by decreasing the solution concentration^33,72^. The obtained surface roughness varies from 50 to 500 nm depending on the laser fluence^70^ and more precise control of etch rate allows higher quality with surface roughness below 5 nm^72,95^. Another example of a high-quality etching process was demonstrated in work^96^, where a novel aqueous solution of Np(SO_3_Na)_3_ was used as the liquid absorber. Zimmer et al. presented a new LIBWE process at a very low etch rate by controlling the thickness of an adsorbed toluene layer^97^. The thin adsorbed layer is formed from an ambient vapor phase. The limited thickness of the adsorbed layer causes the ablation rate saturation and leads to a low etch rate of 1.3 nm/pulse. The etched surface structures show well-defined edges and low surface roughness down to 0.4 nm (RMS). The effect of cavitation bubbles on the surface quality of LIBWE has been studied in work^74^. The decrease of the gap between the transparent substrate and a confined plate (from 10 mm to 0.43 mm) can increase the bubble lifetime and enhance the liquid convection, which leads to the improvement of surface quality.

### Applications of LIBWE

**Fig. 10:**
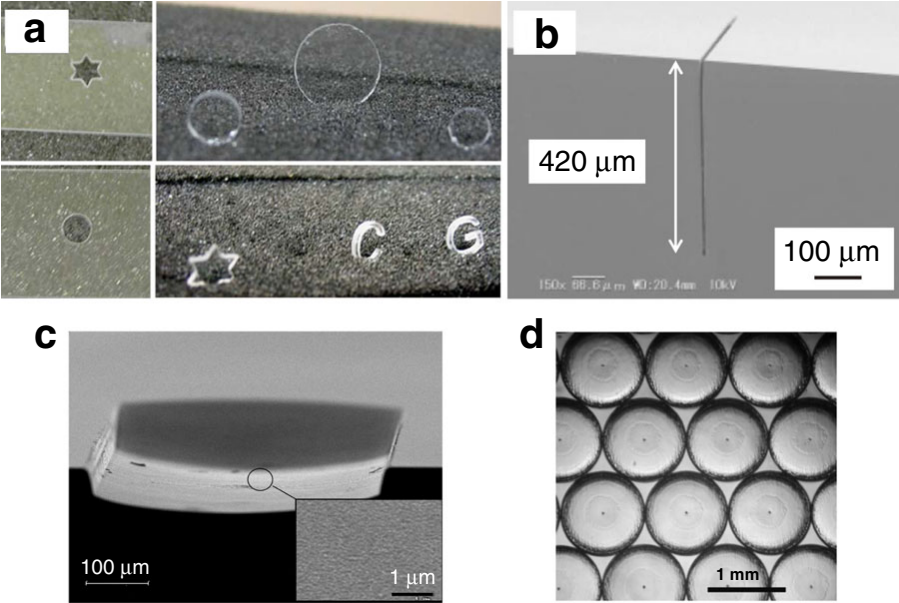
Applications of LIBWE. **a** Different shapes were cut out from a 1-mm-thick glass substrate^73^. **b** High-aspect ratio (up to 60) trench fabricated on silica glass^83^. **c** SEM picture of a cylindrical structure etched into fused silica employing contour mask technique in conjunction with backside etching. The micro-roughness of the surface is below 10 nm RMS at a tech depth of more than 50 μm^77^. **d** Quartz micro-lens array fabricated by LIBWE^84^. Figures reproduced with permission from (**a**) ref. ^74^, © 2020 Elsevier Ltd.; **b** ref. ^81^, Copyright (c)2005 The Japan Society of Applied Physics; **c** ref. ^78^, ©2002 Elsevier Science B.V. and **d** ref. ^93^, ©2007 IOP Publishing Ltd.

LIBWE has been used in a broad range of applications relating to the microprocessing of transparent hard materials: (1) Drilling and cutting. A 1-mm-thick soda-lime glass slide is able to be cut through by a convenient 1064 nm laser using an aqueous CuSO_4_ solution absorber^73^. Figure 10a shows various shapes which were diced out from the glass slides by the LIBWE without suffering any significant cracking. The feasibility of 3D drilling has also been demonstrated by femtosecond laser LIBWE^78,98^. The use of distilled water greatly enhances the removal of the debris and redeposition generated by laser ablation, allowing high-aspect-ratio 3D micro-holes fabrication inside transparent materials. (2) Microstructuring and patterning. In industrial applications, wet etching and plasma etching are the most common methods used in the microstructuring of transparent hard materials. However, both methods require the complicated procedures of patterning and photolithography. LIBWE offers an alternative method to fabricate microstructures/patterns on transparent materials with a single-step process. Defined line-and-space, microfluidic channel, grid and grating structures/patterns by LIBWE have already been demonstrated^75,81,90,94,96,99,100^. The profiles of the fabricated structures show sharp edges and smooth surfaces without debris and micro-cracks. The capability of deep microstructures’ fabrication enables wide applications in the manufacturing of microfluidic channel and high-aspect-ratio trenches^83,100–102^, as shown in Fig. 10b. (3) Micro-optical elements fabrication. Fabrication of these optical structures requires surface roughness < *λ*/20. As shown in Fig. 10c, nanometer-scaled depth control and smooth surface make the LIBWE method capable of directly delivering the required quality without the need for post treatment^77^. The fabrication of Fresnel lens^70^, random phase plate^70^, micro-lens array (Fig. 10d)^84^, and relief grating^82,95^ has been demonstrated on different substrates like fused silica and sapphire. The surface roughness below 5 nm^95,97^ is good enough to enable their practical uses in optical and photonic components.

## Etching-assisted laser micromachining

### Basic principles and characteristics

When conventional laser processing is applied to transparent hard materials, the low accuracy and poor surface roughness largely limit its applicability in optical device fabrication. It is also a great challenge for direct laser processing to realize internal material removal for 3D volume structures’ fabrication. Etching-assisted laser micromachining, which combines the advantages of ultrafast laser machining and wet or dry etching, is considered a potential technology to overcome these challenges. Irradiation of focused ultrafast laser pulses can induce a variety of phase and structural modifications depending on laser fluence and material properties, leaving behind a permanent change in the refractive index or even the material component^103–106^. The laser modification leads to local enhancement of etch rate and facilitates material removal of the modified region under the subsequent etching process. This preferential ablation rate, combined with laser beam scanning in a spatially selective manner, makes it possible to fabricate well-designed 2D even 3D structures on the surface or inside the transparent hard materials. Table 3 provides a summary of the experimental reports on the etching-assisted laser micromachining technology. Both surface and internal laser processing have been extensively studied for the fabrication of a variety of structures and devices, such as 3D micro-channels, micro-lens array, optical waveguide, and integration of multiple components in a single substrate.

**Table 3:** Experimental reports of the etching-assisted laser micromachining technology on different transparent hard materials

Substrate materials	Etching process	Laser wavelength and pulse duration	Fabricated structures and applications	References
Sapphire	Wet	800 nm, 220 fs	3D micro-channels	^36^
	Wet	400 nm, 120 fs	Fresnel zone plate	^125^
	Dry	800 nm, 100 fs	Artificial compound eyes	^133^
	Dry	790 nm, 120 fs	Blazed gratings	^186^
	Wet	400 nm, 120 fs	Dammann grating	^135^
Fused silica	Wet	795 nm, 120 fs	3D micro-channels	^123^
	Wet	800 nm, 30 fs	3D microcoils array	^187,188^
	Wet	800 nm, 100 fs	Integration of optics and micro-mechanics in a single substrate	^147^
	Wet	800 nm, 50 fs	Micro-lens arrays fabrication	^127,130^
	Wet	1030 nm, 380 fs	3D monolithic micro-flexure	^148^
	Wet	800 nm, 100 fs	Optical quality surface fabrication	^128^
	Wet	520 nm, <500 fs	Optofluidic devices	^151^
	Wet	n.s., 270 fs	Opto-mechanical phase modulator	^138^
Foturan glass	Wet	775 nm, 150 fs	3D microstructures	^134^
	Wet	1045 nm, 360 fs	3D optofluidic microchip	^139^
	Wet	775 nm 150 fs	3D integration of micro-optical components	^140^
	Wet	775 nm, 145 ± 5 fs	3D micro-optical components	^149^
YAG crystal	Wet	1030 nm, 350 fs	Diffraction gratings and microstructured optical waveguides	^129^
	Wet	1047 nm, 460 fs	Hollow microstructures	^142^
BK7 glass	Wet	800 nm, 50 fs	Artificial compound eye fabrication	^131^
Silicate glass	Wet	800 nm, 100 fs	Integrated micro/nanofluidic systems	^124^
GaAs	Dry	355 nm, 10 ns	Micro-grating	^126^

*n.s.* not specified

Owing to the wide bandgap, the high absorption of light in transparent materials must be nonlinear (or multiphoton absorption) because there are no allowed electronic transitions at the low single-photon energy^107,108^. For such multiphoton absorption to occur, a typical laser intensity in the order of 10^13^ W/cm^2^ is required^28,107^. This extremely high intensity is generally achieved by tightly focusing an ultrafast laser. Physical processes underlying multiphoton absorption and laser modification have been discussed in a number of works^107,109–111^. When an ultrafast laser pulse with high peak intensity is focused on a transparent material, the excitation of electrons from the valence band to the conduction band is initiated through multiphoton ionization and laser pulse energy is partially transferred to the electrons. The nonlinearly excited electrons are further excited through phonon-mediated linear absorption until they have enough kinetic energy to excite avalanche ionization^27,28^. The highly excited electrons thermalize with the ions and modify the material permanently. The regimes of laser modification are highly dependent on the laser fluence and degree of the electron’s excitation. When the laser fluence is below the self-focusing threshold, non-damage energy depositions with smooth bond energy change can be observed^112,113^. Most of the non-damage energy depositions can lead to the refractive index change of the transparent materials, either positively or negatively^114–119^. By further increasing the laser fluence, a wide variety of phase and structural changes occurs, such as crystalline-to-amorphous phase transition^36^, the formation of nanovoids, and periodic nanogratings^120,121^, and nanocrystallization^122^. After the laser modification, an etching process is performed to selectively remove the modified areas. Both wet and dry etching-assisted laser micromachining of transparent hard materials have been demonstrated^35,103,123^.

### Characteristics of etching-assisted laser micromachining technology

The etching-assisted laser micromachining technology is applicable to almost all transparent materials. Among these, glasses and crystals are commonly used as the substrate materials owing to their high purity and large transparency range. Various micro-devices and components have been demonstrated using glasses and crystals including Foturan glass, sapphire, fused silica, and yttrium aluminum garnet (YAG), as summarized in Table 3.

There are three unique advantages of such an etching-assisted laser micromachining method. First, a prime advantage of this method is the capability of internal laser fabrication on transparent materials, which is a great challenge for other technologies. Second, this method presents high resolution and high surface quality. The combination of a threshold effect of the nonlinear absorption and the negligible thermal diffusion of ultrafast laser pulses enables sub-wavelength resolution which is beyond the diffraction limit^123,124^. In addition, the surface quality can be further improved by the polishing of the subsequent etching process. Typical surface roughness of ~100 nm after laser direct ablation can be reduced to below 40 nm by wet or dry etching processing^125,126^. Third, the laser modification process based on selective laser writing overcomes the need for high-cost masks and complicated lithography procedures^127^, which make this method much simple and cost-effective than the conventional lithographic technique. With these advantages, the etching-assisted laser micromachining method is capable of fabricating complex 2D/3D microstructures with high surface quality on transparent hard materials by a simple process. The researches on this technique mainly focus on the two directions, towards high-quality surface microprocessing and internal microstructures fabrication, which will be discussed below in detail.

### Toward high precision and high surface quality

**Fig. 11:**
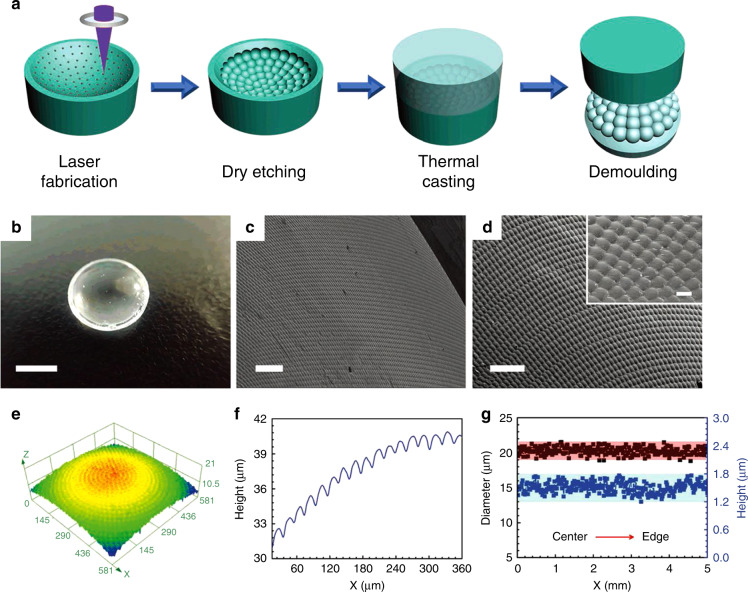
Large area (cm^2^ size) concave compound eyes structures fabricated on a curved K9 glass substrate using laser direct writing followed by dry etching^133^. **a** The schematic diagram of fabrication of sapphire concave compound eyes template and K9 glass compound eye. **b** Photo image (Scale bar: 5 mm), **c** SEM image (Scale bar: 100 μm), and **d** amplified SEM image of K9 glass compound eyes (Scale bar: 100 μm). The inset image in **d** is the local-amplified SEM image of the K9 glass compound eye (Scale bar: 20 μm). **e** 3D and **f** cross-section profiles of the K9 glass compound eye. **g** Diameter and height uniformity of the ommatidia from center to the edge of the macrolens. Reproduced with permission from ref. ^111^, ©2019 WILEY-VCH Verlag GmbH & Co. KGaA, Weinheim

Owning to the nature of nonlinear absorption, the material modification only occurs within the tiny focal volume where the intensity is above the modification threshold. Therefore, femtosecond laser-induced modification can be localized at the center of the focal spot, which could be much smaller than the focused spot size, by adjusting the incident laser intensity. Successive etching is capable to yield microstructures with optical quality and feature size as small as 100 nm by selectively removing the laser-modified regions^128,129^. The surface quality and accuracy of the fabricated devices are good enough to enable their direct use as optical components. Micro-lens array fabrication is one of the successful applications of this technique. Both wet^127,130–132^ and dry^35,133^ etching-assisted laser micromachining have been demonstrated using different transparent hard materials, such as BK7 glass^130^, Foturan glass^134^, silica^127^, and sapphire^133^. An example of micro-lens array fabrication is shown in Fig. 11^133^. Large area (cm^2^ size) concave compound eye structures on curved sapphire substrates have been demonstrated using laser direct writing followed by dry etching. The fabrication efficiency is improved by over two orders of magnitude compared with a traditional point-by-point laser ablation method. The compound eyes show a surface roughness of 1.1 nm (RMS) and excellent optical properties with wide field-of-view imaging and focusing^133^. A shorter wavelength helps to obtain higher machining precision and quality. The fabrication of micro-optical components, such as Dammann grating and Fresnel zone plate with an average roughness of 12 nm have also been demonstrated^125,135^ on sapphire substrates by using the second harmonic generation of an 800 nm femtosecond laser. Etching-assisted laser micromachining is also used to fabricate various microstructures on transparent hard materials for specific functions, such as transparent micro-actuator^136^, high refractive index sensitivity fiber Mach–Zehnder interferometer^137^, and optomechanical phase modulator^138^.

### Towards internal 3D microstructures fabrication

**Fig. 12:**
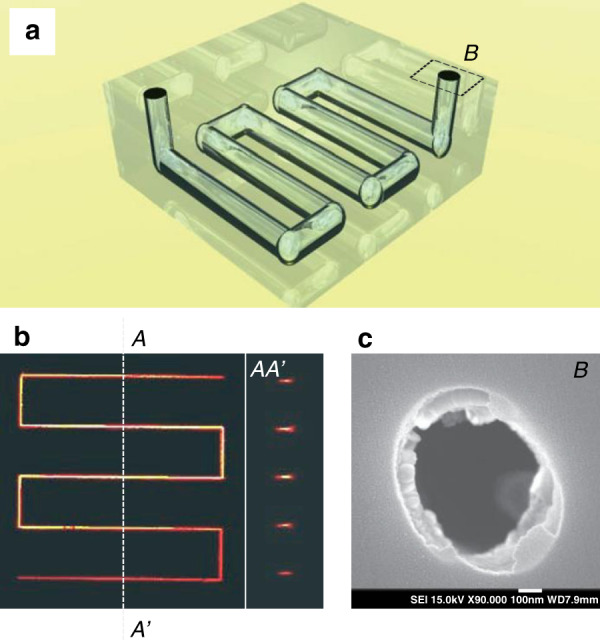
Fabrication of 3D network channels inside sapphire by femtosecond laser writing followed by etching in a 10% HF water solution^36^. **a** The 3D pattern of micro-channels in sapphire. **b** Confocal sectioning of rhodamine photoluminescence in sapphire. The pattern of micro-channels is at a depth of 10 μm. The horizontal length of the channel was 40 μm. **c** SEM image of channel’s cross-section (section B shown in Fig. 12a), scale bar: 100 nm. Reproduced with permission from ref. ^36^, ©2006 WILEY-VCH Verlag GmbH & Co. KGaA, Weinheim

**Fig. 13:**
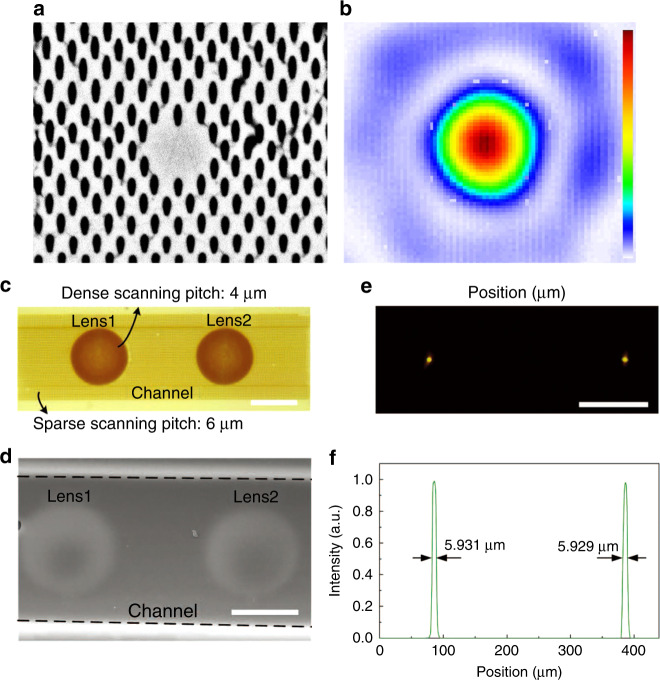
Fabrication of micro-optical devices and integration of multiple components inside transparent materials. **a**, **b** Nanostructured waveguide fabricated in YAG crystal and Diffraction-limited near-field image of the waveguide output mode measured at 1550 nm^129^. **c**, **d** Optical microscopy and SEM images of the glass micro-lens integrated into micro-channel^139^. **e**, **f** Focusing characteristics of the glass micro-lens in a micro-channel in air. Scale bars: 100 μm. Figures reproduced from (**a**, **b**) ref. ^129^, ©2020 Springer Nature Limited; **c**–**f** ref. ^139^, ©2018 WILEY-VCH Verlag GmbH & Co. KGaA, Weinheim

Transparent hard material allows the laser beam to be focused into its bulk substrate without causing surface damage, making it possible to achieve internal laser-induced modification. By combining 3D laser writing and chemical wet etching, 3D microstructures fabrication inside transparent materials has become feasible. The technology was first demonstrated by using silica substrates^123^. Since then, it has been extended to various transparent hard materials, including sapphire^36,129^, Foturan glass^134,139,140^, optical fiber^141^, and YAG crystal^129,142^. The laser-induced modification inside laser crystals can significantly enhance etch rate by more than five orders of magnitude, giving rise to an extremely high etch selectivity up to 10^5^ between the laser modified region and original region^129^. High etch selectivity permits the fabrication of almost 1-cm long through-channels with an aspect ratio of ~200 in fused silica^143^ and 3D network channels without apparent constraints on their length in sapphire^36^, as shown in Fig. 12. These unique capabilities of 3D microprocessing, high etch selectivity and high precision open the possibility for direct fabrication of complex microfluidics^123,134,144–146^, micro-mechanicals^147,148^, and micro-optical devices and components^122,139,140,149^ inside transparent materials, which are difficult to be realized by traditional lithographic technology. Figure 13a presents the demonstration of a nanostructured waveguide fabricated inside YAG with a core size of 1.1 × 1.3 μm^2^, the average pore size of 166 × 386 nm^2^, and length of 4 mm^129^.

Another important application of the etching-assisted laser micromachining technology is that it allows the integration of various functional components into a single glass microchip by a simple procedure, which can find increasing uses in optofluidics, integrated optics, and lab-on-a-chip^13,27,28,41,108,142,150^. An example of the integrated microchip is shown in Fig. 13c–f, where an all-glass 3D optofluidic microchip with built-in micro-lenses is fabricated inside glass microfluidic channels by optimized wet etching-assisted femtosecond laser modification. Yang et al have demonstrated the fabrication of micro-nanofluidic channels buried in silicate glass^124^ with the minimum width of the nanofluidic channel is down to less than 50 nm. The fabricated micro-nanofluidic channels can be applied to DNA analysis. The fabrication of optofluidic integrated cell sorter has also been demonstrated on fused silica substrates^151^. The cell sorter is capable of sorting single cells with optical forces on the basis of their fluorescence. Both fluorescence detection and single-cell sorting functions are integrated into the microfluidic chip by femtosecond laser processing followed by chemical etching.

### Other hybrid laser precision engineering technologies

There are several other hybrid laser technologies for the precision machining of transparent hard materials.

### Laser-induced backside dry etching method

The laser-induced backside dry etching method (LIBDE) is a modified LIBWE where the liquid absorber is substituted by a solid film^152^. Silver, copper, carbon, and aluminum are commonly used as laser energy absorber^153,154^. Usually, the transparent substrate is coated with a thin film with a thickness of 15–120 nm. The film is irradiated by a laser beam from the other side of the substrate. When the laser fluence is above the threshold of the metal layer, it is removed from the irradiated spots and the substrate is etched at the same time due to the recoil effect of the evaporated materials. In principle, transparent hard materials suitable for LIBWE can also be processed by LIBDE. However, LIBDE is proved to be more effective and simpler with a maximum etch rate up to 600 nm/pulse and without the need of a container containing the liquid absorber^155^. The feasibility of grating fabrication on transparent materials has been demonstrated, with the etched grating showing a sub-micrometer period down to 275 nm^152^.

### Laser-induced front side etching

The above-discussed techniques, such as LIPAA, LIBWE, and LIBDE, are applicable in a backside configuration, where the laser beam has to pass through the transparent substrates before the ablation process. This causes restrictions concerning the shape and thickness of the substrate. A flat front surface is generally required with these back-side laser processing techniques.

In the laser-induced front side etching (LIFE), the laser beam is directly focused onto the front side of the transparent substrates, which avoids the issues of absorption and interference from the substrate material^156–158^. With the aim of increasing laser energy absorption, a thin absorber layer is deposited on the front surface, such as chromium^159,160^, aluminum^161^, silver^160^, titanium^160^, and silicon monoxide layer^156^. Comparative studies show that the single-shot ablation thresholds for various glass samples can be reduced by 2 orders of magnitude (from hundreds of J/cm^2^ to a few or tens of J/cm^2^) with the absorber layer^161^. The effect of the absorber layer on LIFE is similar as that in LIBDE^156^. It is rapidly heated and ionized under laser irradiation, thus assists the laser ablation. The LIFE method allows nanometer-precision etching of transparent materials with surface roughness below 10 nm^156,159^, high resolution up to 400 nm^156^ and minimum etching depth down to few nm^157^. The main disadvantage of the LIFE is that an additional step is required to remove the deposited layer after the laser ablation.

### Hybrid laser processing by two lasers

Every single laser has its limitations and advantages during laser processing of transparent hard materials. The combination of two lasers or two processing steps can take full advantage of different lasers and enable both high-speed and high-quality machining of transparent materials^42^. K. Sugioka et al. have demonstrated a two-laser process using *F*_2_ (157 nm) and KrF (248 nm) excimer lasers^26^. Significant improvement of the ablation quality of fused silica was achieved than any single laser processing. The two-laser hybrid processing is also capable of enhancing processing efficiency^162^. The processing efficiency of laser machining of BK7 glasses by two lasers with different pulse widths was increased by 2.3 times that of a single laser^163^. One of the important applications of the two-laser hybrid processing is to fabricate high-quality microfluidic channels and micro-optics by laser processing followed by CO_2_ laser polishing^164^. In general, a nanosecond or femtosecond laser is used to machine a pre-defined structure or geometry on a transparent substrate. It is then treated with a CO_2_ laser to polish the surface^164,165^. Upon irradiation of the CO_2_ laser, the structure surface melts and re-solidifies, resulting in a reduction of roughness. The controlled CO_2_ laser surface polishing has proved to be a practical and valuable alternative to the traditional polishing of glasses due to its abrasive-free and non-contact nature^166–168^. It is shown that the surface roughness Ra can be reduced to optical quality grade from hundreds of nm^169,170^. The fabrication of a variety of micro-devices, such as microfluidic channels^171^, micro-lenticular lens array mold^169^, axicon lens^170^, and high-Q micro-cavities^172^ have been demonstrated on fused silica by this hybrid technique.

### Water-jet guided laser processing

Water-jet guided laser processing is a technology that combines the advantages of laser processing and those of water-jet cutting^173,174^. The laser is guided in the water jet by total internal reflection at the water/air interface in a manner similar to optical fibers. The light-guiding jet hits the sample, which is machined by the laser beam. The water jet also has the functions of cooling the material during the processing and removing the cut debris. Compared with direct laser processing, the use of water jet addresses not only the problem of the heat-affected zone but also other problems like focusing problems, burr formation material deposition. Such a technique has demonstrated a significant reduction of heat and extraordinarily precise cut edges. The main disadvantage of this technology is the low resolution which is limited by the water jet diameter, typically, between 50 and 200 μm^173^. The main applications of the technique focus on cutting, drilling, and ablation of metals and semiconductor wafers^175–177^. The technique can also be used for cutting of transparent hard materials, such as sapphire, SiC, and GaN^178–180^. The efficient cutting of sapphire up to 3 mm thick has been demonstrated^180^. The cut samples exhibit parallel walls with a roughness <0.5 μm and a kerf width of <100 μm. Green et al.^178^ studied the dicing of SiC wafers by the water jet-guided laser. It is shown that the cutting speed is improved by 40% than that of abrasive sawing while maintaining a high cut quality with no damage and no contamination to the substrates.

### Conclusions and outlook

Laser processing of transparent hard materials remains a great challenge in terms of material absorption, surface quality, and crack formation. Hybrid laser engineering can overcome these challenges and exhibit unique capabilities for the precision micromachining of transparent hard materials with much simpler procedures and greater flexibility than conventional techniques. Rapid progress has been made in applying these hybrid techniques for microstructuring, patterning, cutting, color marking of transparent hard materials, and even direct fabrication of micro-devices and optical components at the feature size of ~100 nm and low surface roughness below 20 nm. The etching-assisted laser micromachining also offers the ability to fabricate hollow 3D microstructures and micro-channels with various geometries inside transparent substrates, which holds great promise for the integration of microfluidic, micro-mechanical, and micro-optical components in a single chip with a simple process. Table 4 provides a comparison of these techniques in terms of their overall performances and applications in the processing of transparent hard materials. It is shown that each of the hybrid techniques has particular characteristics, strengths, and weaknesses, and therefore it is critical to select the best method for a given application and material.

**Table 4:** Comparison of the hybrid laser processing techniques

Hybrid techniques	Regimes of hybrid	Surface roughness	Feature size	Etch/ablation rate	Surface/internal	Applications
LIPAA	Plasma-assisted	100 nm (ns laser)^62^	14–30 μm^49^	1–45 nm/pulse^49^	Surface	Patterning, cutting, structuring
		18.1 nm (fs laser)^54^	17.3–40 μm^45^	0.48 μm/pass^45^		
LIBWE	Absorptive liquid-assisted	0.4–500 nm^70,97^	0.53–4 μm^78,82^	0–650 nm/pulse^92,93,184^	Both	Etching, cutting, structuring
EALM	Post etching process	1.1–12 nm^125,133^	<50^124,129^	100 μm/h^129^	Both	3D structures, micro-components
LIFE	Plasma-assisted	5–10 nm^156,159^	<350 nm^157^	A few to 800 nm/pulse^156^	Surface	Etching, structuring
LIBDE	Backside dry etching	<10 nm^155^	~600 nm/pulse^155^	275 nm^152^	Surface	Etching, cutting, structuring
Two-laser processing	Two lasers	9.5–34 nm^169,170^	1 μm^170^	NA	Surface	Microstructuring, micro-components
Water-jet guided	Water jet assisted	<0.5 μm^180^	30–80 μm^180^	0.1–9 mm/s^179,180^	Surface	Cutting

*EALM* etching-assisted laser micromachining, *NA* not applicable

With the technical maturity and the increasingly wide applications of transparent hard materials in consumer electronics and mobile devices, the research attention on hybrid laser precision engineering will focus more on performance improvement and extension of application fields in the future. On the performance side, there is still a lot of room for improvement in terms of fabrication resolution, efficiency, and surface quality. Although some hybrid laser micromachining methods, such as dual-beam femtosecond laser LIPAA, LIBWE using an adsorbed toluene layer and etching-assisted laser micromachining, can achieve the fabrication of microstructures with high resolution and quality which meet the requirements of optical components, the surface roughness is still much higher than those obtained by traditional mechanical polishing methods. Moreover, the laser processing quality varies greatly from material to material and a certain process is only applicable to a specific material. More efforts are required to further study the physical mechanisms behind the hybrid laser technologies for different materials under diverse conditions. A deeper understanding of these mechanisms will help to improve the processing quality by precisely manipulating the processing parameters. There is a trade-off between laser fabrication quality and efficiency. To a certain extent, high-quality laser processing is often achieved at the expense of efficiency. Parallel laser processing shows a great potential to address this contradiction^181^. It can be combined with the hybrid laser micromachining and enables high-quality laser processing at high productivity.

As technology continues to advance, hybrid laser precision engineering should also continue to find more innovative applications. First, the hybrid laser engineering technologies can be extended their application to the silicon substrate. Silicon is opaque to visible light but transparent to IR light. This feature opens the window to use hybrid laser technologies for the micromachining of silicon substrates. For example, wet etching-assisted laser micromachining is able to realize the fabrication of complex 3D structures deep inside silicon chips which current lithography methods cannot achieve^182^. The hybrid laser micromachining techniques will find increasing applications in silicon-based devices fabrication, such as non-planar and embedded electronic–photonic devices, and integration of these devices into one chip. Second, these years have witnessed a rapid growth in the applications of diamond in the fields of machining tools, microelectronics, and optical elements due to its impressive combination of physical, chemical, and mechanical properties. However, there are very few studies that utilize hybrid laser technologies to process diamond substrates. Third, in the fields of flat-panel display and portable electronic devices, new applications of transparent hard materials continue to emerge, such as patterned sapphire LED substrate, micro-lens, and lens array for smartphone and 3D glass in organic LED. There is a growing demand for laser structuring and cutting of transparent and heterogeneous materials with high quality and accuracy. Hybrid laser micromachining can play an important role and more efforts should be made to improve the applicability and convenience of these methods based on practical application needs.

## References

[1] Isberg J (2004). Single crystal diamond for electronic applications. Diam. Relat. Mater..

[2] Berman LE (1993). Diamond crystal X-ray optics for high-power-density synchrotron radiation beams. Nucl. Instrum. Methods Phys. Res. Sect. A.

[3] Koizumi S (2001). Ultraviolet emission from a diamond pn junction. Science.

[4] Akselrod MS, Bruni FJ (2012). Modern trends in crystal growth and new applications of sapphire. J. Cryst. Growth.

[5] Khattak CP (2016). World’s largest sapphire for many applications. J. Cryst. Growth.

[6] Dobrovinskaya, E. R., Lytvynov, L. A. & Pishchik, V. *Sapphire: Material, Manufacturing, Applications*. (Springer, New York, 2009).

[7] Götze J (2009). Chemistry textures and physical properties of quartz-geological interpretation and technical application. Mineral. Mag..

[8] Rahim K, Mian A (2017). A review on laser processing in electronic and MEMS packaging. J. Electron. Packag..

[9] Dubey AK, Yadava V (2008). Laser beam machining—a review. Int. J. Mach. Tools Manuf..

[10] Cheng J (2013). A review of ultrafast laser materials micromachining. Opt. Laser Technol..

[11] Lan B (2004). Laser precision engineering of glass substrates. Jpn. J. Appl. Phys..

[12] Yang J (2014). Design and fabrication of broadband ultralow reflectivity black Si surfaces by laser micro/nanoprocessing. Light.

[13] Sugioka K, Cheng Y (2012). Femtosecond laser processing for optofluidic fabrication. Lab a Chip.

[14] Kerse C (2016). Ablation-cooled material removal with ultrafast bursts of pulses. Nature.

[15] Liu HG (2019). Self-organized periodic microholes array formation on aluminum surface via femtosecond laser ablation induced incubation effect. Adv. Funct. Mater..

[16] Chryssolouris, G. *Laser Machining: Theory and Practice*. (Springer Science & Business Media, 2013).

[17] Faisal, N. et al. Laser micromachining of engineering materials—a review. in *Micro and Nano Machining of Engineering Materials* (eds Kumar, K., Zindani, D. & Kumari, N.) Part III (Springer, Cham, 2019).

[18] Majumdar JD, Manna I (2011). Laser material processing. Int. Mater. Rev..

[19] Wang H (2017). On-chip laser processing for the development of multifunctional microfluidic chips. Laser Photonics Rev..

[20] Samant AN, Dahotre NB (2009). Laser machining of structural ceramics—a review. J. Eur. Ceram. Soc..

[21] Chong TC, Hong MH, Shi LP (2010). Laser precision engineering: from microfabrication to nanoprocessing. Laser Photonics Rev..

[22] Vorobyev AY, Guo CL (2013). Direct femtosecond laser surface nano/microstructuring and its applications. Laser Photonics Rev..

[23] Ihlemann J (2003). Fabrication of submicron gratings in fused silica by F_2_-laser ablation. Appl. Phys. A.

[24] Gallais L, Cormont P, Rullier JL (2009). Investigation of stress induced by CO_2_ laser processing of fused silica optics for laser damage growth mitigation. Opt. Express.

[25] Herman, P. R. et al. Processing applications with the 157-nm fluorine excimer laser. *Proc. SPIE 2992, Excimer Lasers, Optics, and Applications*. (SPIE, Jose, CA, United States, 1997).

[26] Sugioka K (2003). Hybrid laser processing for microfabrication of glass. Appl. Phys. A.

[27] Sugioka K, Cheng Y (2014). Ultrafast lasers-reliable tools for advanced materials processing. Light.

[28] Gattass RR, Mazur E (2008). Femtosecond laser micromachining in transparent materials. Nat. Photonics.

[29] Amina (2019). Ionization behavior and dynamics of picosecond laser filamentation in sapphire. Optoelectron. Adv..

[30] Wlodarczyk KL (2018). Rapid laser manufacturing of microfluidic devices from glass substrates. Micromachines.

[31] Hwang DJ (2009). Self-guided glass drilling by femtosecond laser pulses. Appl. Phys. A.

[32] Zhang J, Sugioka K, Midorikawa K (1998). Laser-induced plasma-assisted ablation of fused quartz using the fourth harmonic of a Nd^+^:YAG laser. Appl. Phys. A.

[33] Wang J, Niino H, Yabe A (1999). One-step microfabrication of fused silica by laser ablation of an organic solution. Appl. Phys. A.

[34] Ito Y (2017). High-efficiency and precision cutting of glass by selective laser-assisted milling. Precis. Eng..

[35] Liu XQ (2017). Dry-etching-assisted femtosecond laser machining. Laser Photonics Rev..

[36] Juodkazis S (2006). Control over the crystalline state of sapphire. Adv. Mater..

[37] Phillips KC (2015). Ultrafast laser processing of materials: a review. Adv. Opt. Photonics.

[38] Malinauskas M (2016). Ultrafast laser processing of materials: from science to industry. Light.

[39] Watanabe W, Li Y, Itoh K (2016). [INVITED] Ultrafast laser micro-processing of transparent material. Opt. Laser Technol..

[40] Jiang LJ (2016). Femtosecond laser direct writing in transparent materials based on nonlinear absorption. MRS Bull..

[41] Osellame R (2011). Femtosecond laser microstructuring: an enabling tool for optofluidic lab‐on‐chips. Laser Photonics Rev..

[42] Nisar S, Li L, Sheikh MA (2013). Laser glass cutting techniques—a review. J. Laser Appl..

[43] Hanada, Y., Sugioka, K. & Midorikawa, K. Laser-induced plasma-assisted ablation (LIPAA): fundamental and industrial applications. in *Proc. SPIE 6261, High-Power Laser Ablation VI*. (SPIE, Taos NM, US, 2006).

[44] Hong, M. H. et al. Crack-free laser processing of glass substrate and its mechanisms. in *Proc. SPIE 4637, Photon Processing in Microelectronics and Photonics*. (SPIE, San Jose, California, United States, 2002).

[45] Liu HG (2020). High-aspect-ratio crack-free microstructures fabrication on sapphire by femtosecond laser ablation. Opt. Laser Technol..

[46] Pan CF (2017). Fabrication of micro-texture channel on glass by laser-induced plasma-assisted ablation and chemical corrosion for microfluidic devices. J. Mater. Process. Technol..

[47] Malhotra R (2013). Laser-induced plasma micro-machining (LIPMM) for enhanced productivity and flexibility in laser-based micro-machining processes. CIRP Ann..

[48] Zhang J, Sugioka K, Midorikawa K (1998). High-speed machining of glass materials by laser-induced plasma-assisted ablation using a 532-nm laser. Appl. Phys. A.

[49] Zhang J, Sugioka K, Midorikawa K (1999). High-quality and high-efficiency machining of glass materials by laser-induced plasma-assisted ablation using conventional nanosecond UV, visible, and infrared lasers. Appl. Phys. A.

[50] Hong, M. H. et al. Laser-induced-plasma-assisted ablation for glass microfabrication. in *Proc. SPIE 4595, Photonic Systems and Applications*. (SPIE, Singapore, Singapore, 2001).

[51] Chao H (2012). Laser induced backside wet and dry etching of solar glass by short pulse ytterbium fiber laser irradiation. J. Laser Appl..

[52] Rahman TU (2020). Enhancement of pulsed laser-induced silicon plasma-assisted quartz ablation by continuous wave laser irradiation. J. Laser Appl..

[53] Saxena I, Ehmann KF (2014). Multimaterial capability of laser induced plasma micromachining. J. Micro Nano-Manuf..

[54] Li Y, Liu HG, Hong MH (2020). High-quality sapphire microprocessing by dual-beam laser induced plasma assisted ablation. Opt. Express.

[55] Singh, J. P. & Thakur, S. N. *Laser-Induced Breakdown Spectroscopy*. 2nd edn. (Elsevier, Amsterdam, 2020).

[56] Noack J, Vogel A (1999). Laser-induced plasma formation in water at nanosecond to femtosecond time scales: calculation of thresholds, absorption coefficients, and energy density. IEEE J. Quantum Electron..

[57] Hanada Y (2005). Double-pulse irradiation by laser-induced plasma-assisted ablation (LIPAA) and mechanisms study. Appl. Surf. Sci..

[58] Hanada Y (2006). Transient electron excitation in laser-induced plasma-assisted ablation of transparent materials. J. Appl. Phys..

[59] Hong, M. H. et al. Optical diagnostics in laser-induced plasma-assisted ablation of fused quartz. in *Proc. SPIE 4088, First International Symposium on Laser Precision Microfabrication*. (SPIE, Omiya, Saitama, Japan, 2000).

[60] Hong MH (2002). Laser microfabrication of transparent hard materials and signal diagnostics. Appl. Surf. Sci..

[61] Zhang J, Sugioka K, Midorikawa K (1998). Direct fabrication of microgratings in fused quartz by laser-induced plasma-assisted ablation with a KrF excimer laser. Opt. Lett..

[62] Jaber, H., Binder, A. & Ashkenasi, D. High-efficiency microstructuring of VUV window materials by laser-induced plasma-assisted ablation (LIPAA) with a KrF excimer laser. in *Proc. SPIE 5339, Photon Processing in Microelectronics and Photonics III*. (SPIE, San Jose, CA, United States, 2004).

[63] Sugioka K (2003). Advanced materials processing based on interaction of laser beam and a medium. J. Photochem. Photobiol. A.

[64] Jiang W (2017). High contrast patterning on glass substrates by 1064 nm pulsed laser irradiation. Optical Mater. Express.

[65] Hanada Y (2007). Colour marking of transparent materials by laser-induced plasma-assisted ablation (LIPAA). J. Phys..

[66] Xu SJ (2017). Ultrafast fabrication of micro-channels and graphite patterns on glass by nanosecond laser-induced plasma-assisted ablation (LIPAA) for electrofluidic devices. J. Mater. Process. Technol..

[67] Lu XZ (2017). Laser-induced-plasma-assisted ablation and metallization on C-plane single crystal sapphire (c-Al_2_O_3_). Micromachines.

[68] Lee JM, Jang JH, Yoo TK (2000). Scribing and cutting a blue LED wafer using a Q-switched. Nd:YAG laser. Appl. Phys. A.

[69] Verhoff B, Harilal SS, Hassanein A (2012). Angular emission of ions and mass deposition from femtosecond and nanosecond laser-produced plasmas. J. Appl. Phys..

[70] Kopitkovas G (2003). Fabrication of micro-optical elements in quartz by laser induced backside wet etching. Microelectron. Eng..

[71] Sun XY (2019). Study on ablation threshold of fused silica by liquid-assisted femtosecond laser processing. Appl. Opt..

[72] Zimmer K, Braun A, Böhme R (2003). Etching of fused silica and glass with excimer laser at 351 nm. Appl. Surf. Sci..

[73] Huang ZQ (2008). Laser etching of glass substrates by 1064 nm laser irradiation. Appl. Phys. A.

[74] Xie XZ (2013). Cavitation bubble dynamics during laser wet etching of transparent sapphire substrates by 1064 nm laser irradiation. J. Laser Micro/Nanoeng..

[75] Ding XM (2003). Laser-induced backside wet etching of sapphire. Jpn. J. Appl. Phys..

[76] Long JY (2019). Incubation effect during laser-induced backside wet etching of sapphire using high-repetition-rate near-infrared nanosecond lasers. Opt. Laser Technol..

[77] Böhme R, Braun A, Zimmer K (2002). Backside etching of UV-transparent materials at the interface to liquids. Appl. Surf. Sci..

[78] Li Y (2001). Three-dimensional hole drilling of silica glass from the rear surface with femtosecond laser pulses. Opt. Lett..

[79] Hwang DJ, Choi TY, Grigoropoulos CP (2004). Liquid-assisted femtosecond laser drilling of straight and three-dimensional microchannels in glass. Appl. Phys. A.

[80] Ehrhardt M (2010). Microstructuring of fused silica by laser-induced backside wet etching using picosecond laser pulses. Appl. Surf. Sci..

[81] Xu J (2015). Vertical sidewall electrodes monolithically integrated into 3D glass microfluidic chips using water-assisted femtosecond-laser fabrication for in situ control of electrotaxis. RSC Adv..

[82] Pissadakis S, Böhme R, Zimmer K (2007). Sub-micron periodic structuring of sapphire by laser induced backside wet etching technique. Opt. Express.

[83] Kawaguchi Y (2005). Etching a micro-trench with a maximum aspect ratio of 60 on silica glass by *laser-induced backside wet etching* (LIBWE). Jpn. J. Appl. Phys..

[84] Kopitkovas G (2007). Laser induced backside wet etching: mechanisms and fabrication of micro-optical elements. J. Phys..

[85] Kawaguchi Y (2005). Transient pressure induced by laser ablation of toluene, a highly laser-absorbing liquid. Appl. Phys. A.

[86] Böhme R, Zimmer K (2005). The influence of the laser spot size and the pulse number on laser-induced backside wet etching. Appl. Surf. Sci..

[87] Wang J, Niino H, Yabe A (2000). Micromachining of transparent materials with super-heated liquid generated by multiphotonic absorption of organic molecule. Appl. Surf. Sci..

[88] Vass C (2004). Experiments and numerical calculations for the interpretation of the backside wet etching of fused silica. Thin Solid Films.

[89] Xie XZ (2019). Laser machining of transparent brittle materials: from machining strategies to applications. Opto-Electron. Adv..

[90] Niino H (2003). Surface micro-fabrication of silica glass by excimer laser irradiation of organic solvent. J. Photochem. Photobiol. A.

[91] Zimmer K, Ehrhardt M, Böhme R (2010). Simulation of laser-induced backside wet etching of fused silica with hydrocarbon liquids. J. Appl. Phys..

[92] Zimmer K (2006). Backside etching of fused silica with UV laser pulses using mercury. J. Phys. D.

[93] Böhme R, Zimmer K (2007). Indirect laser etching of fused silica: towards high etching rate processing. Appl. Surf. Sci..

[94] Zimmer K, Böhme R (2008). Laser-induced backside wet etching of transparent materials with organic and metallic absorbers. Laser Chem..

[95] Zimmer K (2002). Excimer laser-induced etching of sub-micron surface relief gratings in fused silica using phase grating projection. Appl. Phys. A.

[96] Ding X (2002). Laser-induced high-quality etching of fused silica using a novel aqueous medium. Appl. Phys. A.

[97] Zimmer K, Böhme R, Rauschenbach B (2004). Laser etching of fused silica using an adsorbed toluene layer. Appl. Phys. A.

[98] Li Y, Qu SL (2013). Water-assisted femtosecond laser ablation for fabricating three-dimensional microfluidic chips. Curr. Appl. Phys..

[99] Yasui Y (2002). Microetching of fused silica by laser ablation of organic solution with XeCl excimer laser. Appl. Surf. Sci..

[100] Tan YX (2019). Water-assisted laser drilling of high-aspect-ratio 3D microchannels in glass with spatiotemporally focused femtosecond laser pulses. Optical Mater. Express.

[101] Huang ZQ (2007). Quality glass processing by laser induced backside wet etching. J. Laser Micro/Nanoeng..

[102] Kwon KK (2020). High aspect ratio channel fabrication with near-infrared laser-induced backside wet etching. J. Mater. Process. Technol..

[103] Liu XQ (2019). Etching-assisted femtosecond laser modification of hard materials. Opto-Electron. Adv..

[104] Tan DZ (2016). Femtosecond laser induced phenomena in transparent solid materials: fundamentals and applications. Prog. Mater. Sci..

[105] Krol DM (2008). Femtosecond laser modification of glass. J. Non-Crystalline Solids.

[106] Itoh K (2006). Ultrafast processes for bulk modification of transparent materials. MRS Bull..

[107] Schaffer CB, Brodeur A, Mazur E (2001). Laser-induced breakdown and damage in bulk transparent materials induced by tightly focused femtosecond laser pulses. Meas. Sci. Technol..

[108] Sugioka K (2014). Femtosecond laser 3D micromachining: a powerful tool for the fabrication of microfluidic, optofluidic, and electrofluidic devices based on glass. Lab a Chip.

[109] Stuart BC (1995). Laser-induced damage in dielectrics with nanosecond to subpicosecond pulses. Phys. Rev. Lett..

[110] Stuart BC (1996). Nanosecond-to-femtosecond laser-induced breakdown in dielectrics. Phys. Rev. B.

[111] Bloembergen N (1997). A brief history of light breakdown. J. Nonlinear Optical Phys. Mater..

[112] Beresna M, Gecevičius M, Kazansky PG (2014). Ultrafast laser direct writing and nanostructuring in transparent materials. Adv. Opt. Photonics.

[113] Chan JW (2001). Structural changes in fused silica after exposure to focused femtosecond laser pulses. Opt. Lett..

[114] Rodenas A, Kar AK (2011). High-contrast step-index waveguides in borate nonlinear laser crystals by 3D laser writing. Opt. Express.

[115] Liu JR (2004). Waveguide shaping and writing in fused silica using a femtosecond laser. IEEE J. Sel. Top. Quantum Electron..

[116] Calmano T (2010). Nd:YAG waveguide laser with 1.3 W output power, fabricated by direct femtosecond laser writing. Appl. Phys. B.

[117] Li QK (2017). Multilevel phase-type diffractive lens embedded in sapphire. Opt. Lett..

[118] Bhardwaj VR (2005). Femtosecond laser-induced refractive index modification in multicomponent glasses. J. Appl. Phys..

[119] Reupert A (2020). Angular scattering pattern of femtosecond laser‐induced refractive index modifications in optical fibers. Adv. Optical Mater..

[120] Kanehira S (2005). Periodic nanovoid structures via femtosecond laser irradiation. Nano Lett..

[121] Shimotsuma Y (2003). Self-organized nanogratings in glass irradiated by ultrashort light pulses. Phys. Rev. Lett..

[122] Cheng Y (2003). Optical gratings embedded in photosensitive glass by photochemical reaction using a femtosecond laser. Opt. Express.

[123] Marcinkevičius A (2001). Femtosecond laser-assisted three-dimensional microfabrication in silica. Opt. Lett..

[124] Liao Y (2013). Direct laser writing of sub-50 nm nanofluidic channels buried in glass for three-dimensional micro-nanofluidic integration. Lab a Chip.

[125] Li QK (2016). Sapphire-based Fresnel zone plate fabricated by femtosecond laser direct writing and wet etching. IEEE Photonics Technol. Lett..

[126] Yang SN (2020). Periodic microstructures fabricated by laser interference with subsequent etching. Nanomaterials.

[127] Chen F (2010). Maskless fabrication of concave microlens arrays on silica glasses by a femtosecond-laser-enhanced local wet etching method. Opt. Express.

[128] Sikorski Y (2006). Fabrication and characterization of microstructures with optical quality surfaces in fused silica glass using femtosecond laser pulses and chemical etching. Appl. Opt..

[129] Ródenas A (2019). Three-dimensional femtosecond laser nanolithography of crystals. Nat. Photonics.

[130] Tong SY (2014). Large-scale high quality glass microlens arrays fabricated by laser enhanced wet etching. Opt. Express.

[131] Deng ZF (2016). Dragonfly-eye-inspired artificial compound eyes with sophisticated imaging. Adv. Funct. Mater..

[132] Chen F (2014). Rapid fabrication of a large-area close-packed quasi-periodic microlens array on BK7 glass. Opt. Lett..

[133] Liu XQ (2019). Rapid engraving of artificial compound eyes from curved sapphire substrate. Adv. Funct. Mater..

[134] Masuda M (2003). 3-D microstructuring inside photosensitive glass by femtosecond laser excitation. Appl. Phys. A.

[135] Li QK (2016). Sapphire-based Dammann gratings for UV beam splitting. IEEE Photonics J..

[136] Lenssen B, Bellouard Y (2012). Optically transparent glass micro-actuator fabricated by femtosecond laser exposure and chemical etching. Appl. Phys. Lett..

[137] Sun XY (2015). A robust high refractive index sensitivity fiber Mach–Zehnder interferometer fabricated by femtosecond laser machining and chemical etching. Sens. Actuators A.

[138] Casamenti, E. et al. Optomechanical suspended waveguide for broadband phase modulation with frequency memory effect. Preprint at *arXiv* https://arxiv.org/abs/1906.02035 (2019).

[139] Hu YL (2018). All-glass 3D optofluidic microchip with built-in tunable microlens fabricated by femtosecond laser-assisted etching. Adv. Optical Mater..

[140] Wang ZK, Sugioka K, Midorikawa K (2007). Three-dimensional integration of microoptical components buried inside photosensitive glass by femtosecond laser direct writing. Appl. Phys. A.

[141] Haque M (2014). Chemical-assisted femtosecond laser writing of lab-in-fibers. Lab a Chip.

[142] Choudhury D (2013). Three-dimensional microstructuring of yttrium aluminum garnet crystals for laser active optofluidic applications. Appl. Phys. Lett..

[143] Kiyama S (2009). Examination of etching agent and etching mechanism on femotosecond laser microfabrication of channels inside vitreous silica substrates. J. Phys. Chem. C.

[144] Bellouard Y (2004). Fabrication of high-aspect ratio, micro-fluidic channels and tunnels using femtosecond laser pulses and chemical etching. Opt. Express.

[145] Cheng Y, Sugioka K, Midorikawa K (2004). Microfluidic laser embedded in glass by three-dimensional femtosecond laser microprocessing. Opt. Lett..

[146] Gottmann J (2017). Selective laser-induced etching of 3D precision quartz glass components for microfluidic applications—up-scaling of complexity and speed. Micromachines.

[147] Bellouard Y, Said AA, Bado P (2005). Integrating optics and micro-mechanics in a single substrate: a step toward monolithic integration in fused silica. Opt. Express.

[148] Tielen V, Bellouard Y (2014). Three-dimensional glass monolithic micro-flexure fabricated by femtosecond laser exposure and chemical etching. Micromachines.

[149] Cheng Y (2003). Three-dimensional micro-optical components embedded in photosensitive glass by a femtosecond laser. Opt. Lett..

[150] Malek CGK (2006). Laser processing for bio-microfluidics applications (part I). Anal. Bioanal. Chem..

[151] Bragheri F (2012). Optofluidic integrated cell sorter fabricated by femtosecond lasers. Lab a Chip.

[152] Hopp B (2006). Production of submicrometre fused silica gratings using laser-induced backside dry etching technique. J. Phys. D.

[153] Smausz T (2007). Influence on the laser induced backside dry etching of thickness and material of the absorber, laser spot size and multipulse irradiation. Appl. Surf. Sci..

[154] Böhme R, Zimmer K, Rauschenbach B (2006). Laser backside etching of fused silica due to carbon layer ablation. Appl. Phys. A.

[155] Hopp B, Vass C, Smausz T (2007). Laser induced backside dry etching of transparent materials. Appl. Surf. Sci..

[156] Ihlemann J (2008). Micro patterning of fused silica by laser ablation mediated by solid coating absorption. Appl. Phys. A.

[157] Lorenz P, Ehrhardt M, Zimmer K (2012). Laser-induced front side etching: an easy and fast method for sub-μm structuring of dielectrics. Phys. Proc..

[158] Wlodarczyk KL (2016). Direct CO_2_ laser-based generation of holographic structures on the surface of glass. Opt. Express.

[159] Lorenz P, Ehrhardt M, Zimmer K (2012). Laser-induced front side etching of fused silica with KrF excimer laser using thin chromium layers. Phys. Status Solidi A.

[160] Lorenz P (2012). Laser-induced front side etching of fused silica with XeF excimer laser using thin metal layers. Appl. Surf. Sci..

[161] Nieto D (2017). Aluminum thin film enhanced IR nanosecond laser-induced frontside etching of transparent materials. Opt. Lasers Eng..

[162] Kondratenko VS (2020). Glass cutting technology combining two different lasers. Glass Ceram..

[163] Pan YX (2016). Millisecond laser machining of transparent materials assisted by a nanosecond laser with different delays. Opt. Lett..

[164] Temple PA, Lowdermilk WH, Milam D (1982). Carbon dioxide laser polishing of fused silica surfaces for increased laser-damage resistance at 1064 nm. Appl. Opt..

[165] Zhao, L. et al. Rapid CO_2_ laser processing technique for fabrication of micro-optics and micro-structures on fused silica materials. *Proc. Inst. Mech. Eng. Part B*10.1177/0954405420937534 (2020).

[166] Laguarta F, Lupon N, Armengol J (1994). Optical glass polishing by controlled laser surface-heat treatment. Appl. Opt..

[167] Nowak KM, Baker HJ, Hall DR (2006). Efficient laser polishing of silica micro-optic components. Appl. Opt..

[168] Jung S, Lee PA, Kim BH (2016). Surface polishing of quartz-based microfluidic channels using CO_2_ laser. Microfluid. Nanofluid..

[169] Kim C (2014). Fabrication of a fused silica based mold for the microlenticular lens array using a femtosecond laser and a CO_2_ laser. Optical Mater. Express.

[170] Schwarz S (2018). Fabrication of a high-quality axicon by femtosecond laser ablation and CO_2_ laser polishing for quasi-Bessel beam generation. Opt. Express.

[171] Serhatlioglu M (2016). CO_2_ laser polishing of microfluidic channels fabricated by femtosecond laser assisted carving. J. Micromech. Microeng..

[172] Lin JT (2012). On-chip three-dimensional high-Q microcavities fabricated by femtosecond laser direct writing. Opt. Express.

[173] Richerzhagen, B. et al. Water-jet-guided laser processing. in *Proc. SPIE 4830, Third International Symposium on Laser Precision Microfabrication*. (SPIE, Osaka, Japan, 2003).

[174] Tabie VM (2019). Water-jet guided laser cutting technology-an overview. Lasers Manuf. Mater. Process..

[175] Porter JA (2007). Cutting thin sheet metal with a water jet guided laser using various cutting distances, feed speeds and angles of incidence. Int. J. Adv. Manuf. Technol..

[176] Richerzhagen B (2001). Chip singulation process with a water-jet guided laser. Solid State Technol..

[177] Marimuthu S (2019). Water-jet guided laser drilling of SiC reinforced aluminium metal matrix composites. J. Composite Mater..

[178] Green, S., Perrottet, D. & Richerzhagen, B. Damage-free dicing of SiC wafers by water-jet-guided laser. in *Proc. CS MANTECH Conference*. (Vancouver, British Columbia, Canada, 2006).

[179] Nilsson, T. et al. Scribing of GaN wafer for white LED by water-jet-guided laser. in *Proc. SPIE 5366, Light-Emitting Diodes: Research, Manufacturing, and Applications VIII*. (SPIE, San Jose, CA, United States, 2004).

[180] Richmann A (2014). Laser microjet^©^ cutting of up to 3 mm thick sapphire. ICALEO.

[181] Li Y, Hong MJ (2020). Parallel laser micro/nano-processing for functional device fabrication. Laser Photonics Rev..

[182] Tokel O (2017). In-chip microstructures and photonic devices fabricated by nonlinear laser lithography deep inside silicon. Nat. Photonics.

[183] Xing YQ (2020). Assessment machining of micro-channel textures on PCD by laser-induced plasma and ultra-short pulsed laser ablation. Opt. Laser Technol..

[184] Zimmer K (2006). Backside laser etching of fused silica using liquid gallium. Appl. Phys. A.

[185] Spagnolo M (2020). Resonant opto-mechanical modulators and switches by femtosecond laser micromachining. Opt. Express.

[186] Liu, X. Q. et al. Wear-resistant blazed gratings fabricated by etching-assisted femtosecond laser lithography. *J. Lightw. Technol.*10.1109/JLT.2021.3066976 (2021).

[187] Shan C (2015). High-level integration of three-dimensional microcoils array in fused silica. Opt. Lett..

[188] He SG (2012). Facile fabrication of true three-dimensional microcoils inside fused silica by a femtosecond laser. J. Micromech. Microeng..

